# Ensemble attribute profile clustering: discovering and characterizing groups of genes with similar patterns of biological features

**DOI:** 10.1186/1471-2105-7-147

**Published:** 2006-03-16

**Authors:** JR Semeiks, A Rizki, MJ Bissell, IS Mian

**Affiliations:** 1 Life Sciences Division (MS 977-225A), Lawrence Berkeley National Laboratory, 1 Cyclotron Road, Berkeley, CA 94720, USA; 2 Life Sciences Division (MS 74-197), Lawrence Berkeley National Laboratory, 1 Cyclotron Road, Berkeley, CA 94720-8265, USA

## Abstract

**Background:**

Ensemble attribute profile clustering is a novel, text-based strategy for analyzing a user-defined list of genes and/or proteins. The strategy exploits annotation data present in gene-centered corpora and utilizes ideas from statistical information retrieval to discover and characterize properties shared by subsets of the list. The practical utility of this method is demonstrated by employing it in a retrospective study of two non-overlapping sets of genes defined by a published investigation as markers for normal human breast luminal epithelial cells and myoepithelial cells.

**Results:**

Each genetic locus was characterized using a finite set of biological properties and represented as a vector of features indicating attributes associated with the locus (a gene attribute profile). In this study, the vector space models for a pre-defined list of genes were constructed from the Gene Ontology (GO) terms and the Conserved Domain Database (CDD) protein domain terms assigned to the loci by the gene-centered corpus LocusLink. This data set of GO- and CDD-based gene attribute profiles, vectors of binary random variables, was used to estimate multiple finite mixture models and each ensuing model utilized to partition the profiles into clusters. The resultant partitionings were combined using a unanimous voting scheme to produce consensus clusters, sets of profiles that co-occured consistently in the same cluster. Attributes that were important in defining the genes assigned to a consensus cluster were identified. The clusters and their attributes were inspected to ascertain the GO and CDD terms most associated with subsets of genes and in conjunction with external knowledge such as chromosomal location, used to gain functional insights into human breast biology. The 52 luminal epithelial cell markers and 89 myoepithelial cell markers are disjoint sets of genes. Ensemble attribute profile clustering-based analysis indicated that both lists contained groups of genes with the functional properties of membrane receptor biology/signal transduction and nucleic acid binding/transcription. A subset of the luminal markers was associated with metabolic and oxidoreductase activities, whereas a subset of myoepithelial markers was associated with protein hydrolase activity.

**Conclusion:**

Given a set of genes and/or proteins associated with a phenomenon, process or system of interest, ensemble attribute profile clustering provides a simple method for collating and sythesizing the annotation data pertaining to them that are present in text-based, gene-centered corpora. The results provide information about properties common and unique to subsets of the list and hence insights into the biology of the problem under investigation.

## Background

Recent advances in biomedical technologies such as high-throughput molecular profiling provide the ability to identify large numbers of molecules of interest simultaneously. An accessible and straightforward solution to elucidating why a set of genes might be associated with a given phenomenon is manual inspection of the scientific literature and examination of Web-based resources. PubMed [1] is the most widely-used method for accessing the MEDLINE corpus of abstracts and full text articles. Gene-centered corpora such as LocusLink [2], Entrez Gene [3], SGD [4], Wormbase [5], and Flybase [6] are non-redundant, comprehensive, text-based catalogs of information designed to support research on genes and gene families (conserved domains), variation, gene expression, genome annotation, and so on. A "document" in such a corpus corresponds to a genetic locus and includes annotations obtained by automated computational methods and/or manual data curation. Despite the ready availability of multiple biomedical corpora, the task of collating and synthesizing information relevant to a collection of genes remains both a challenging and time-consuming endeavor. Consequently, increasing attention is being paid to developing techniques and tools able to transform data interpretation into a more systematic and automated process (see, for example, [7-11]).

A variety of approaches have been proposed to address the problem of determining the common function(s)specified by a group of genes. The free-text of PubMed abstracts known to be associated with specific genes have been analyzed using concepts from statistical natural language processing (NLP) (see, for example, [12]). Frequently, entries in gene-centered corpora contain annotations for proteins in the form of controlled vocabulary terms from the Gene Ontology (GO) molecular function, cellular component and biological process aspects [13]. Given the GO annotations associated with a list of genes, methods for finding significant shared GO terms and for visualizing the collection include GoMiner [14], GOTermFinder [15], FunSpec [16], GOStat [17], and FuncAssociate [18]. However, such approaches do not exploit other types of annotations that may be assigned to loci. For example, a LocusLink entry can indicate which domains in the Conserved Domain Database (CDD) have been identified in the protein.

This work describes ensemble attribute profile clustering, a novel text-based strategy that employs ideas from statistical information retrieval (IR) and annotation data from gene-centered corpora to discover and characterize groups of genes in a list with shared properties. Whereas NLP focuses on the syntax and semantics of text, statistical IR emphasizes vector space models and probabilistic models for document representation, retrieval and analysis [19,20]. A gene attribute profile is a vector space representation of a genetic locus in which an element of the vector specifies a functional, structural or other property of the locus (an "attribute"). A profile is created from descriptors that can be ascribed to loci such as the molecular weight of an encoded RNA or protein, the hydrophobic content of a protein, whether a transcription factor regulates a gene, a term from a controlled vocabulary, and so on. This study exploits ongoing large-scale annotation efforts and the extensive body of knowledge present in existing resources to facilitate and simplify the task of constructing gene attribute profiles. Here, the profiles consider only the protein product of a locus. In particular, the choice of attributes is restricted to terms in the GO and CDD vocabularies and the terms associated with specific genes are derived from the corresponding LocusLink entries.

The task of ascertaining genes in a list with shared biological properties is posed as one of clustering gene attribute profiles, *i.e.*, determining homogeneous groups of profiles such that profiles in the same cluster are more similar to each other than they are to ones in other clusters. The problem of discovering groups of genes with similar patterns of attributes can be addressed using methods and principles that have been employed to find groups of transcript profiles with related patterns of expression. Simple probabilistic graphical models, finite mixture models in particular, have been utilized to cluster vector space representations of transcript abundances (gene expression profiles) [21-23] and text documents [19]. Multiple runs of a single clustering algorithm or runs of different algorithms on the same data set can yield distinct partitionings so efforts to ameliorate this disparity seek to determine subsets of objects (genes) whose behavior is consistent across the partitionings. Methods proposed for gene expression profiles include re-sampling the data over the algorithm [24] and combining the output from correlation-based clustering techniques [25]. Gene expression profiles can be clustered using distance-based methods because every feature is of the same type (real-valued variables or multinomial variables if the abundances are quantized). The advantage of clustering gene attribute profiles using a model-based approach is that in a finite mixture model, the variable corresponding to a feature has its own probability model meaning that different types of attributes (discrete, multinomial, real-valued, and so on) can be combined and considered within the same framework.

To illustrate the practical utility of ensemble attribute profile clustering, the method was employed in a retrospective study of data extracted from a published transcript profiling study of normal human breast tissue. This prior investigation identified two non-overlapping sets of genes, one list specified markers for luminal epithelial cells and the other markers for myoepithelial cells [26]. For each list of genes, a set of gene attribute profiles was built using the GO and CDD terms assigned to genetic loci by LocusLink. The profiling data were used to estimate four independent finite mixture models and the four partitionings of the profiles produced using these probabilistic models were combined via a unanimous voting scheme. Genes that co-occurred in all four partitionings were identified and attributes that characterized each resultant consensus cluster were determined. Examination of these automatically generated results in conjunction with external knowledge indicated that the two lists contained groups of genes with common biological functions (membrane receptor biology/signal transduction, nucleic acid binding/transcription) and unique functions (oxidoreductase/metabolic activity, hydrolase activity). Thus, although the two collections of genes designated as markers for luminal and myoepithelial breast cells are disjoint sets [26], the encoded proteins specify a limited number of broad functional categories, some of which are shared by both normal human breast tissue cell types. The palette of functions and protein domains highlighted by this study can guide the design of experiments aimed at improving understanding of breast biology. Despite limitations in GO- and CDD-based gene attribute profiles and the approach itself, the results described here demonstrate that ensemble attribute profile clustering can assist in elucidating relationships within and between user-defined sets of genes and/or proteins.

## Results

### Gene attribute profiles for normal human breast tissue-related genes

Ensemble attribute profiling was used to analyze two lists of genes extracted from an extant study of normal human breast tissues and defined by the investigators as markers for luminal epithelial cells and myoepithelial cells [26]. The vector space representation of a gene devised here focused on the structural and functional properties of encoded proteins. In particular, the gene attribute profiles associated with a list of genes were derived from the GO and CDD terms assigned to the loci by the gene-centered corpus LocusLink. Each resultant profile was a vector of binary random variables, *i.e.*, the value of a feature was "1" if the corresponding attribute was associated with the gene and "0" otherwise. The data set constructed from the published list of luminal epithelial cell markers consisted of 52 genes represented as 43-dimensional vectors (the LUMINAL collection). The data set for the myoepithelial cell markers consisted of 89 96-dimensional vectors (the MYOEPITHELIAL collection). For each data set, four independent finite mixture models were estimated and a unanimous voting scheme was employed to combine the four partitionings of the profiles produced by these models. In both cases, every gene could be assigned to a consensus cluster. The influential attributes for the consensus clusters were determined and the results integrated with external knowledge.

### Properties common and unique to proteins encoded by genes in the LUMINAL and MYOEPITHELIAL collections

Two sets of results reports were generated and examined to gain insights into the differences and similarities between the LUMINAL and MYOEPITHELIAL collections. These reports are provided as Additional Files and contain full descriptions of the consensus clusters, gene-specific GO/CDD terms, and annotations present in LocusLink entries but not used as attributes to construct gene attribute profiles. Inspection of these reports showed that in contrast to GO and CDD terms, very few of the genes considered in this study were annotated with KEGG pathways by LocusLink. However, every gene was annotated with a cytoband position (its chromosomal location). Tables 1 and 2 summarize the information on consensus clusters and their influential attributes found in the results reports.

In order to delineate broad functional categories associated with the 52 genes in the LUMINAL collection, the influential and most frequent attributes for each consensus cluster were determined and summarized. These genes were partitioned into four groups, with each group representing a broad functional category (Table 1; Additional Files 1 and 2). The prominent features of the proteins encoded by the genes in the four consensus clusters were as follows: 0 (24 genes), membrane proteins with receptor and/or signal transduction activity; 1 (20 genes), metabolic activity; 2 (4 genes), nucleic acid binding and transcription; and 3 (4 genes), oxidoreductase and ion transport activity. The 89 genes in the MYOEPITHELIAL collection were partitioned into four groups, three of which represented broad functional categories (Table 2; Additional Files 3 and 4). The prominent features of the proteins encoded by the genes in the four consensus clusters were as follows: 0 (48 genes), intracellular proteins; 1 (17 genes), membrane proteins with receptor and/or signal transduction activity; 2 (15 genes), protein hydrolase activity; and 3 (9 genes), nucleic acid binding and transcription.

The properties common to proteins encoded by genes in the LUMINAL and MYOEPITHELIAL collections were integral membrane proteins that are receptors and/or involved in signal transduction, and proteins involved in nucleic acid binding and transcription. The LUMINAL genes were distinguished by a subset of proteins with metabolic and oxidoreductase activity, whereas protein hydrolase activity was a distinctive feature of a subset of the MYOEPITHELIAL genes. Although the 52 LUMINAL genes and 89 MYOEPITHELIAL genes were mutually exclusive, the resulting functional clusters specified a limited number of high-level functions. Furthermore, some of these functions were associated with both gene lists, and hence both cell types.

The absence of influential CDD attributes in summary Tables 1 and 2 can be explained by the relative paucity of CDD terms compared to GO terms in a LocusLink entry, and different aspects of the process used to create and cluster gene attribute profiles. For the LUMINAL collection, no CDD term was assigned to three or more genes, so feature selection resulted in profiles composed exclusively of GO term attributes (Summary section of Additional File 2). Although the MYOEPITHELIAL collection profiles contained GO and CDD term attributes (Additional File 4), no CDD term was assigned to half the genes in a consensus cluster so no CDD term was designated an influential attribute (Additional File 3).

Examination of the CDD term attributes in the profiles used for clustering showed that four protein domains were each associated with three MYOEPITHELIAL genes but no LUMINAL gene. These MYOEPITHELIAL unique domains were as follows, (i) "Helix-loop-helix domain, found in specific DNA-binding proteins that act as transcription factors" (cd00083) was associated with ID2 (inhibitor of DNA binding 2), ID3 (inhibitor of DNA binding 3), and MYC (v-myc myelocytomatosis viral oncogene homolog); (ii) "Calcium-binding EGF-like domain, present in a large number of membrane-bound and extracellular (mostly animal) proteins. Many of these proteins require calcium for their biological function and calcium-binding sites have been found to be located at the N-terminus of particular EGF-like domains" (cd00054) was associated with SELE (selectin E), FAT2 (FAT tumor suppressor homolog 2), and JAG1 (jagged 1, Alagille syndrome); (iii) "Cadherin EGF LAG seven-pass G-type receptor [Signal transduction mechanisms]" (KOG4289) was associated with SELE, PCDHGA12 (protocadherin gamma subfamily A, 12), and CDH13 (adherin 13, H-cadherin); and (iv) "Cadherin repeat domain" (cd00031) was associated with FAT2, PCDHGA12, and CDH13. Each of these protein domains represents a superfamily of diverse functions, so further studies using a variety of sequence analysis tools will be required to produce fine-grained information about the specific domain subfamily represented in the MYOEPITHELIAL gene list.

The output produced by ensemble attribute profile clustering should be viewed as a useful synthesis of the annotations assigned by gene-centered corpora with the results warranting additional examination. For example, of the genes discussed above, CDH13, FAT2, JAG1, PCDHGA12 and SELE are present in MYOEPITHELIAL consensus cluster 1 so a simple interpretation of the functional biology linking them is a connection to membrane biology (Table 2). Indeed, all are assigned the GO term "integral to membrane" (Additional Files 3 and 4). However, closer inspection of all GO terms indicates that, for example, CDH13 is an adhesion molecule ("homophilic cell adhesion") whereas JAG1 is a ligand for the receptor Notch ("Notch binding", "Notch signaling pathway").

Although the pathways in which a protein is involved are important properties of a gene, their exclusion from the vector space model formulated here appears to have had minimal impact on the results described above. Inspection of the KEGG pathways assigned to genes in a consensus cluster largely reinforced the observations made by examination of influential GO and CDD attributes. In LUMINAL consensus cluster 3, for example, the GO molecular function term "oxidoreductase activity" was an influential attribute and three of the four genes were assigned the KEGG pathway "Oxidative phosphorylation". For other gene lists, it may be necessary to include KEGG pathway attributes in the gene attribute profiles used as input for consensus clustering. In general, the issue is not only a matter of whether certain properties should be included as attributes in the vector space representation of a gene, but also whether the relevant information is present in and hence can be extracted from text-based, gene-centered corpora.

## Discussion

Ensemble attribute profile clustering-based analysis of two disjoint lists of breast tissue-related genes demonstrates the utility of this general purpose strategy for discovering and characterizing groups of genes with similar patterns of features. The approach separates the tasks of computing clusters of profiles and determining attributes important in defining each cluster. Thus, one enhancement would be to replace the finite mixture model used to perform one-way probabilistic clustering with a two-way clustering algorithm that estimates groups of genes and groups of attributes simultaneously (see, for example, [27]). An alternative approach might be model-based subspace clustering in which clusters of objects are identified based on subsets of attributes [28,29]. Ensemble attribute profile clustering bears some resemblance to TXTGate, an existing framework for analyzing a group of genes that is based on clustering and vector space representations [30]. The two methods are distinguished by their choice of features in the vector space models, the algorithm used for clustering (hierarchical versus probabilistic), and the absence of cluster combination in TXTGate. Irrespective of the algorithm used for clustering, the major determinants of the biological insights that can be deduced from examination of (consensus) clusters and (influential) attributes are the choice of attributes used to construct gene attribute profiles, the ability to encode functionally important properties as features, and the independence of attributes in the vector space model representation of a gene.

Since a particular choice of attributes embodies prior knowledge about the properties of genes deemed to be most important, vector space models can be tailored to emphasize different desired aspects. The advantages of employing GO and CDD terms to build profiles is their capacity to capture a broad range of concepts in structural, molecular and cellular biology, the widespread adoption of terms in these controlled vocabularies to annotate genes, and the ability to extract these descriptors for an arbitrary collection of genes from gene-centered corpora. For the LUMINAL and MYOEPITHELIAL collections, the GO and CDD terms appear sufficient to encapsulate the knowledge that would have been added by incorporation of KEGG pathway-based attributes. However, the GO and CDD vocabularies are unable in general to capture information such as normal versus aberrant phenotypes and aspects related to evolutionary biology. To ameliorate the former limitation, genes could be characterized using terms that were binary random variables indicating association with disease classes such as "immune", "metabolic", "cancer", "cardiovascular", "aging", "development", "infection", "neurodegeneration" and so on. However, the consensus clusters could differ because genes would be grouped on the basis of similar patterns of phenotypic properties rather than the protein structural and functional properties examined in this work.

Although GO and CDD terms could be augmented with attributes related to phenotypes, the ensuing profiles would remain partial descriptions of genes. To highlight and illustrate this issue, the inability of GO- and CDD-based profiles to capture knowledge about genomic context was investigated. This topic was selected because of increasing interest in the relationship between co-ordinated gene expression and gene clustering in eukaryotes (for a recent review, see [31]). The chromosomal positions of genes in the LUMINAL and MYOEPITHELIAL collections are given in Additional File 5. Although most LUMINAL and MYOEPITHELIAL genes are separated by large physical distances and/or many interlopers, some are separated by four or fewer genes. LUMINAL genes meeting this criterion are: RARRES3 – [1 interloper] -HRASLS3 (11q12.3). MYOEPITHELIAL genes meeting this criterion are FAT2 – [0] – SPARC (5q33.1); MT3 – [2] – MT1K – [2] – MT1B – [0] – MT1F – [0] – MT1G – [1] – MT1X (16ql2.2); S100A7 – [4] – S100A2 (Iq21.3); and NLGN2 – [6] – POLR2A – [4] – EIF4A1 (17pl3.1). Further analysis and studies of the genomic regions containing these blocks of genes could reveal critical regulatory elements, a hypothesis that could not have been formulated from inspection of clusters of GO- and CDD-based gene attribute profiles.

Attributes related to genomic context could be generated by creating a series of binary variables defining the window within which a gene could occur, for example, cytogenetic location such as "Iq", "1", "2q", and "3p.l3", fixed physical distance such as 10 kbp, and region coinciding with a BAC clone. However, the optimal window size is an open question since too small a bin would result in a large increase in the number of attributes to be added to the vector, too large a bin might be uninformative, and different regions of the genome might require different sizes depending on local factors such as gene density.

In both the "bag-of-words" representation of documents used commonly in IR and the "bag-of-attributes" representation of genes considered here, the features in a vector are assumed to be unrelated to each other and the order in which they occur is not significant (exchangeability). However, dependencies amongst and between GO and CDD terms means that this assumption of attribute independence is violated in GO- and CDD-based profiles. The directed acyclic graph underpinning the GO vocabulary defines semantic relationships amongst GO terms so that, for example, the term intracellular is a parent of nucleus. The co-occurrence of protein domains is likely to be non-random because, for example, a nucleic acid binding domain is more likely to occur in conjunction with a nuclease domain than with some other domain. Finally, the GO molecular function terms assigned to a gene are related to the presence of specific protein domains in the encoded protein, for example, DNA binding is associated with zinc finger domains.

## Conclusion

Despite the limitations in ensemble attribute profile clustering discussed above, analysis of genes associated with two normal human breast tissue cell types using GO- and CDD-based gene attribute profiles demonstrates that the strategy provides a simple but useful method for synthesizing prior biological knowledge and facilitating the extraction of new insights. No specific gene is present in both the list of epithelial cell markers and the list of myoepithelial cell markers [26]. However, examination of the consensus clusters discovered and characterized for the LUMINAL and MYOEPITHELIAL collections indicates that both collections contain genes encoding membrane proteins (receptors, signal transducers), and proteins involved in nucleic acid binding and transcription. In addition to these common properties are biological functions that appear to be unique. Four of the 52 LUMINAL genes encode proteins with metabolic and oxidoreductase activity; fifteen of the 89 MYOEPITHELIAL genes are associated with protein hydrolase activity.

Improved understanding of the biology of the breast requires integrating data and information from multiple sources as well as studies of both normal and aberrant breast tissue. The two gene lists analyzed here were the outcome of a cDNA microarray study of luminal and myoepithelial cells isolated from primary cultures of reduction mammoplasty specimens [26]. Elsewhere, SAGE technology was used to identify markers for the same two cell types isolated from freshly dissected normal breast reduction tissue [32]. Finally, a comparative genomic hybridization (CGH) analysis of 43 grade III invasive ductal breast carcinomas positive for basal cytokeratin 14, and 43 grade- and age-matched CK14-negative controls found significant differences in CGH profiles between these two groups in terms of mean number of changes and types of chromosomal alterations [33]. Table 3 lists genes common to the two transcript profiling studies and of these, those located in regions found to be altered in the CGH study. These genes, notably ones marked with †, encode proteins associated with membrane biology, transcription, and (protein) metabolism. Thus, they represent good candidates for experiments aimed at predicting disease outcome and elucidating the molecules and pathways involved in normal breast function.

## Methods

### Markers for normal human breast luminal and myoepithelial cells

A published transcript profiling study of two cell types found in normal human breast tissue and obtained from breast-reduction surgery defined two non-overlapping lists of genes: 76 markers for luminal epithelial cells and 131 markers for myoepithelial cells [26]. Each of these pre-defined lists was analyzed using ensemble attribute profile clustering.

### Ensemble attribute profile clustering

#### Data conversion

A set of sequence IDs was mapped to entries in the gene-centered corpus LocusLink [34] (the methods described here can be applied readily to Entrez Gene [3], the successor to LocusLink). Each ID was translated to a unique entry in the NCBI Reference Sequence database (RefSeq) [35] and the RefSeq matched to its corresponding LocusID (a single genetic locus). To accomplish this mapping quickly and easily, a custom SQL (PostgreSQL 8) database was built to consolidate biological sequence annotations from several sources including LocusLink and RefSeq. A Perl script utilizing the Bioperl [36] LocusLink parser was written to load the LocusLink LL_tmpl file obtained from the NCBI FTP server into this database. For the July 2004 build of LocusLink, 52 of the 76 luminal epithelial cell IDs and 89 of the 131 myoepithelial cell IDs could be mapped to LocusIDs. These *N *= 52 and *N *= 89 LocusIDs ("genes") were designated the LUMINAL and MYOEPITHELIAL collections respectively. The list of human breast tissue-related genes in each collection was analyzed as described below.

#### Feature generation

The gene attribute profiles associated with a collection of *N *genes were based on the GO [37] and CDD [38] terms found in the LocusLink entries specified by the *N *LocusIDs. A Perl script was written to assemble these profiles by querying the SQL database described above. For each gene, this script collated the following attributes, (i) the explicit GO and CDD terms in the gene's LocusLink entry, and (ii) all GO terms that were parents of the explicit GO terms as implied by the structure of the GO directed acyclic graph (DAG). A GO term was discarded if it belonged to the set deemed to be uninformative, namely cellular_component, molecular_function, biological_process, cellular_component unknown, molecular_function unknown, biological_process unknown, obsolete cellular component, obsolete molecular function, obsolete biological process, and the Cellular Component term unlocalized.

The non-redundant set of GO and CDD terms formed from the union of *N *gene-specific terms provided the palette of attributes used to characterize genes. In the vector space model representation of a gene considered here, the significance of a feature was quantified by treating each attribute as a binary random variable with "1" signifying that the GO/CDD term was associated with a gene and "0" otherwise (the order of features in the vector was not significant). For a particular gene, the weight of a GO term was "1" if it was an explicit GO term for that gene, or if it was an ancestor in the GO DAG of an explicit GO term. The weight of a CDD term was "1" if it was an explicit CDD term (although a domain may occur multiple times in a protein, the CDD term is listed only once in a LocusLink entry). The weights assigned to attributes depend upon the completeness of information present in the gene-centered corpus used to construct the profiles. Given the limitations of such resources and the evolving nature of biomedical research, a weight of "0" was viewed more as an indicator of an absence of knowledge about association with a gene rather than the presence of definitive information about a lack of association.

#### Feature selection

In the feature generation script described above, some of the features in the *N *feature vectors produced by the preceding steps were eliminated because the GO or CDD terms were assigned to a small number of genes. For the two gene lists examined here and eight others, a number of attributes were associated with only a few genes (data not shown). In each case, the simple heuristic of retaining attributes assigned to at least three genes was found to provide a parsimonious approach to identifying attributes likely to be informative. If *P *is the number of features that remain after application of all the preceding feature generation and selection steps, the data set used for subsequent clustering was *N *P-dimensional vector space representations of genes. For the LUMINAL and MYOEPITHELIAL collections, there were 52 43-dimensional and 89 96-dimensional gene attribute profiles respectively.

#### Model-based clustering

A data set of *N *P-dimensional gene attribute profiles was used to estimate a simple probabilistic model, a finite mixture model, where each of the *P *binary random variables was modelled using a Bernouilli distribution. The specific implementation of finite mixture models utilized here, AutoClass C version 3.3.4 [39], used a Bayesian approach to determine the number of clusters *K *that best fit the data. Learning a model from data entailed determining *K *(model selection) and the probability parameters of each cluster (parameter estimation). AutoClass addressed the combinatorial optimization problem via a search over the space of models and parameters using the Expectation Maximization (EM) algorithm. Starting from a random initialization, the iterative search procedure partitioned the data into clusters (classes) and adjusted the parameters to find their (local) maximum likelihood estimates. This open-ended procedure was continued until a convergence criterion was met. The profiles were not smoothed to account for unobserved events (GO/CDD terms not being assigned to genes by LocusLink as a result of incomplete biological knowledge). AutoClass was run from a series of small high-level Perl scripts that also implemented the cluster combination and report-generation steps described below. The scripts transformed the feature matrix output by the feature generation script into the proper AutoClass input format. The following non-default AutoClass parameter settings were used: max_duration = 10800, max_cycles = 500, start_j_list = 40,45, and force_new_search_p = true. A trained *K *-class AutoClass model was employed to calculate the probability of a profile having been generated by each of the *K *clusters.

#### Cluster combination

To find a consistent partitioning of the profiles, the results from multiple finite mixture models were integrated to produce consensus clusters. A given clustering algorithm can produce different partitions of the same data set depending upon its assumptions about the data, and choices such as the distance metric in tree-based methods. With AutoClass, different initializations can yield distinct numbers of clusters and assignments of data points to clusters. Combining many classifiers, each a weak learner whose strengths and limitations may differ from the others, can produce a system where the prediction performance of the ensemble is competitive with approaches that focus on estimating a single, good classifier (reviewed in [40]). However, strategies for fusing different clustering algorithms and/or their results have received less attention [41-43].

The simple approach used here was based on four independent finite mixture models estimated using a given data set. For each trained model, a disjoint partitioning of the profiles was produced by assigning each gene to the cluster which maximized its probability (a hard assignment). Consensus clusters were identified using a unanimous voting scheme in which pairs of profiles that co-occurred in all partitionings were placed in the same group by the scripts. To implement this voting algorithm, the problem of finding co-occurring pairs of genes in several different AutoClass runs was restated as the problem of finding consistently-comprised components in different graphs. Given this reinterpretation, a part of the voting algorithm was implemented using the standard C++ Boost Graph library [44] using Perl scripts as a high-level interface.

All genes in the LUMINAL and MYOEPITHELIAL collections could be equated with a consensus cluster, although this outcome is not guaranteed by the voting algorithm. Although further studies are required to devise methods for selecting the optimal number of partitionings to combine and the voting scheme itself, experiments indicated that the choice of four independent AutoClass models and a unanimous voting scheme yielded consensus clusters that were relatively consistent (data not shown). Increasing the number of partitionings but retaining unanimous voting produced more consensus clusters, each with fewer genes. With a majority voting scheme in which genes needed to co-occur in only three of the four partitionings, the result was consensus clusters whose makeup was largely unchanged together with reassignment of a small number of genes.

#### Cluster interpretation

To elucidate the shared properties of genes in a consensus cluster, attributes important in defining the cluster were determined and the results inspected in conjunction with external knowledge. To assist in interpreting consensus clusters, two complementary reports were generated automatically using a Perl script and the SQL annotation database. The first report gave the annotation counts and relative frequencies of all attributes associated with genes in each consensus cluster. The second report provided a number of other annotations for a gene found in LocusLink: gene symbol, GO and CDD terms, cytoband, and physiological pathways from the Kyoto Encyclopedia of Genes and Genomes (KEGG) database [45].

For the purpose of analysis, a set of "influential" attributes was defined for each consensus class. For a consensus cluster with two or more genes, influential attributes were defined as those attributes assigned to at least half of the genes assigned in that consensus cluster. For a consensus cluster with two genes, influential attributes were those assigned to both genes.

### Chromosomal positions of genes

The chromosomal positions of genes in the LUMINAL and MYOEPITHELIAL collections were determined as follows. Every LocusID for a gene was mapped to its RefSeq and the RefSeq mapped to chromosomal coordinates in the May 2004 UCSC build of the Golden Path database, specifically, the maximal transcription start and end coordinates in the table refFlat [46]. These coordinates were used to calculate the spacing between the genes in terms of both number of base pairs and number of intervening RefSeq accessions (interlopers).

## Authors' contributions

J.R.S. wrote the software and performed the experiments. A.R. and M.J.B. analyzed the data. I.S.M. conceived the study and analyzed the data. All authors wrote and approved the manuscript.

## Supplementary Material

Additional File 1 Information on the 52 genes in the LUMINAL collection. For each gene, the "LocusID", "Symbol", "Name", "Cytoband Location", "GO Terms", "Domain Terms", "KEGG Pathway" and "OMIM" fields contain data taken from the July 2004 release of LocusLink. Genes are grouped by their assigned consensus clusters and ordered by their cytoband locations within each cluster. The GO and CDD terms shown are attributes in the gene attribute profiles used as input for probablistic clustering (explicit terms assigned to a gene by LocusLink plus implicit GO terms). The attributes marked with an asterisk are influential attributes, GO/CDD terms that occur with a frequency > 0.5 in clusters with two or more genes. Click here for file

Additional File 2 Information on the GO and CDD attributes associated with the consensus clusters discovered for genes in the LUMINAL collection. For each consensus cluster, the file shows the number of genes assigned to the cluster, along with the absolute count and relative frequency of all attributes associated with the cluster's genes. The section labeled "Summary" shows all the attributes associated with genes in the data set, ordered by average count across all consensus clusters. Click here for file

Additional File 3 Information on the 89 genes in the consensus clusters discovered and characterized for the MYOEPITHELIAL collection, along with their associated GO and CDD attributes. The formats are the same as for luminal-all.html and luminal-attrfreq.html. Click here for file

Additional File 4 Information on the 89 genes in the consensus clusters discovered and characterized for the MYOEPITHELIAL collection, along with their associated GO and CDD attributes. The formats are the same as for luminal-all.html and luminal-attrfreq.html. Click here for file

Additional File 5 Information on the chromosomal locations of the luminal epithelial (brown) and myoepithelial (green) genes. For each gene, the "LocusID" and "Symbol" fields are taken from the July 2004 release of LocusLink. The "Cytoband", "Start" and "End" fields are taken from the May 2004 UCSC assembly of the human genome. Genes are ordered by chromosomal coordinates (p arm followed by q arm). The "Overall" column indicates the distance in kilobases between the adjacent transcription end and start coordinates of the two closest genes from the union of the LUMINAL and MYOEPITHELIAL collections, as well as the number of RefSeqs found in this interval. "pter" and "qter" indicate the number of genes from a telomere to the closest gene in either collection. Click here for file

## Figures and Tables

**Table 1:** Consensus clusters and influential attributes identified for the LUMINAL collection. "Genes" specifies the number of genes in a consensus cluster. "Attributes" lists influential attributes, defined as attributes that occur with a frequency ≥ 0.5 in a consensus cluster with two or more genes, and 1.0 otherwise; †signifies an attribute with frequency 1.0 (consensus cluster 1 lacks influential attributes because no attribute satisfies the frequency criterion, *i.e.*, assignment to 10 or more of the 20 genes). The abbreviations are as follows: CC, GO Cellular Component term; MF, GO Molecular Function term; and BP, GO Biological Process term. Complete information on the genes in the consensus clusters and full lists of attributes can be found in Additional Files 1 and 2.

Cluster no.	Consensus cluster	Gene symbol (LocusID) and attributes
**0**	*24 Genes*	ANXA11 (311); ATP1B1 (481); BACE2 (25825); CD151 (977); CD24 (934); CLDN4 (1364); CX3CL1 (6376); ERBB3 (2065); GADD45B (4616); KLK6 (5653); LCN2 (3934); LGALS3 (3958); MFI2 (4241); MUC1 (4582); NKTR (4820); PLXNB2 (23654); PPAP2C (8612); PRSS8 (5652); PSME1 (5720); RARRES1 (5918); SEMA3B (7869); SEMA6C (10500); SH3BP5 (9467); SLC9A3R1 (9368)
	*Attributes*	CC: cell†; membrane; integral to membrane
**1**	*20 Genes*	CST6 (1474); CTL2 (57153); CUGBP2 (10659); FSTL3 (10272); GLRX (2745); HRASLS3 (11145); JWA (10550); KIAA0220 (283846); KIAA1641 (57730); LGALS9 (3965); MATN4 (8785); MGC11242 (79170); MGLL (11343); NK4 (9235); PCBD (5092); QSCN6 (5768); RARRES3 (5920); SCAP2 (8935); TNFAIP2 (7127); WFDC2 (10406)
	*Attributes*	
**2**	*4 Genes*	BCL11A (53335); GTF3A (2971); UBN1 (29855); ZFP36L1 (677)
	*Attributes*	CC: nucleus†; intracellular† cell†
		MF: nucleic acid binding†; binding†; transcription regulator activity; DNA binding; transition metal ion binding
		BP: regulation of transcription, DNA-dependent ; metabolism
**3**	*4 Genes*	DHCR24 (1718); MTATP6 (4508); MTCO2 (4513); MTND4 (4538)
	*Attributes*	CC: membrane†; intracellular†; cytoplasm†; cell†; mitochondrion; integral to membrane
		MF: catalytic activity†; transporter activity; oxidoreductase activity; hydrogen ion transporter activity
		BP: metabolism†; electron transport

**Table 2:** Consensus clusters and influential attributes identified for the MYOEPITHELIAL collection. The format is the same as Table 1. Complete information on the genes in the consensus clusters and full lists of attributes can be found in Additional Files 3 and 4.

Cluster no.	Consensus cluster	Gene symbol (LocusID) and attributes
**0**	*48 Genes*	AKR1C2 (1646); ARHGAP1 (392); BLCAP (10904); CBR1 (873); COL1A1 (1277); COL5A2 (1290); CSRP2 (1466); CSTA (1475); DKK3 (27122); EIF2B2 (8892); FABP5 (2171); FBL (2091); FLJ20297 (55627); FZD6 (8323); GAPD (2597); GPI (2821); GSTO1 (9446); GSTP1 (2950); HNRPF (3185); ID2 (3398); ID3 (3399); IMPA2 (3613); KRT1 (3848); MT1B (4490); MT1F (4494); MT1G (4495); MT1K (4499); MT1X (4501); MT3 (4504); MTHFD2 (10797); NDRG1 (10397); PES1 (23481); PKM2 (5315); PPFIBP2 (8495); PRDX1 (5052); PRNP (5621); PTHLH (5744); PTRF (284119); S100A2 (6273); S100A7 (6278); SERPINB5 (5268); SERPINH1 (871); SHMT2 (6472); SPARC (6678); SPRR1B (6699); SURF4 (6836); TIMP3 (7078); UQCRFS1 (7386)
	*Attributes*	CC: cell; intracellular
		BP: physiological process
**1**	*17 Genes*	AREG (374); ATP1B3 (483); CAV1 (857); CD44 (960); CDH13 (1012); FAT2 (2196); GPC1 (2817); ITGA3 (3675); ITGA6 (3655); JAG1 (182); LGALS7 (3963); MAEA (10296); NLGN2 (57555); PCDHGA12 (26025); PLP2 (5355); SELE (6401); SLC1A5 (6510)
	*Attributes*	CC: cell†; membrane; integral to membrane; plasma membrane; integral to plasma membrane
		MF: binding
		BP: cellular process; cell communication; physiological process; cell adhesion; cellular physiological process
**2**	*15 Genes*	CCT2 (10576); CSNK1A1 (1452); CTSC (1075); CTSL (1514); DUSP6 (1848); EIF4A1 (1973); IGF1R (3480); MMP3 (4314); MYLK (4638); PLAT (5327); PRSS1 (5644); PRSS11 (5654); RAN (5901); SFN (2810); TIMP1 (7076)
	*Attributes*	MF: catalytic activity; binding; hydrolase activity
		BP: protein metabolism†; physiological process†; metabolism†; cellular process
**3**	*9 Genes*	EPAS1 (2034); KHSRP (8570); MYC (4609); POLR2A (5430); SNAI2 (6591); TBX3 (6926); TP73L (8626); TRIM28 (10155); TRIM29 (23650)
	*Attributes*	CC: intracellular†; cell†; nucleus
		MF: nucleic acid binding†; DNA binding†; binding†; transcription regulator activity; transcription factor activity
		BP: transcription, DNA-dependent†; physiological process†; metabolism†; regulation of transcription, DNA-dependent; regulation of transcription; transcription from Pol II promoter

**Table 3:** Differentially expressed genes common to two independent transcript profiling studies of human breast tissue [26,32]. The table shows LUMINAL and MYOEPITHELIAL genes [26] that were identified also by a SAGE study [32]; the column labelled "Cluster" indicates the "Consensus cluster" to which the gene was assigned (taken from Table 1 or Table 2). †denotes genes present in genomic regions found to be altered in a CGH study of invasive ductal breast carcinomas [33].

LUMINAL		
Cluster	Location	Symbol (LocusID): description

**0**	*6q21*	CD24 (934): CD24 antigen, small cell lung carcinoma cluster 4 antigen
	*†7q11.23*	CLDN4 (1364): claudin 4
	*12q13*	ERBB3 (2065): v-erb-b2 erythroblastic leukemia viral oncogene homolog 3 (avian)
	*†17q25.2*	SLC9A3R1 (9368): solute carrier family 9 (sodium/hydrogen exchanger), isoform 3 regulator 1
	*†19pl3.3*	GADD45B (4616): growth arrest and DNA-damage-inducible, *β*
**1**	*14q32*	TNFAIP2 (7127): tumor necrosis factor, *α*-induced protein 2
**2**	*14q22-q24*	ZFP36L1 (677): zinc finger protein 36, C3H type-like 1

MYOEPITHELIAL		

Cluster	Location	Symbol (LocusID): description

**0**	*2p25*	ID2 (3398): inhibitor of DNA binding 2, dominant negative helix- loop- helix protein
	*5q31.3-q32*	SPARC (6678): secreted protein, acidic, cysteine-rich, osteonectin
	*12p13*	GAPD (2597): glyceraldehyde-3-phosphate dehydrogenase
	*†17q21.3-q22.1*	COL1A1 (1277): collagen, type I, *α *1
**1**	*1q22-q25*	SELE (6401): selectin E, endothelial adhesion molecule 1
	*† 4q13-q21*	AREG (374): amphiregulin, schwannoma- derived growth factor
**2**	*1p35.3*	SFN (2810): stratifin
	*† 10q26.3*	PRSS11 (5654): protease, serine, 11 [IGF binding]
**3**	*2p21-p16*	EPAS1 (2034): endothelial PAS domain protein 1
	*3q27-q29*	TP73L (8626): tumor protein p73-like

## References

[B1] PubMed. http://www.ncbi.nlm.nih.gov/entrez/query.fcgi?db=PubMed.

[B2] LocusLink. http://www.ncbi.nlm.nih.gov/LocusLink.

[B3] Entrez Gene. http://www.ncbi.nlm.nih.gov/entrez/query.fcgi?db=gene.

[B4] SGD. http://www.yeastgenome.org.

[B5] Wormbase. http://www.wormbase.org.

[B6] Flybase. http://www.flybase.org.

[B7] MacCallum R, Kelley R, Steinberg M (2000). SAWTED: Structure Assignment With Text Description – Enhanced detection of remote homologues with automated SW1SS-PROT annotation comparisons. Bioinformatics.

[B8] Jenssen T, Laegreid A, Komorowski J, Hovig E (2001). A literature network of human genes for high-throughput analysis of gene expression. Nature Genetics.

[B9] Raychaudhuri S, Chang J, Imam F, Altman R (2003). The computational analysis of scientific literature to define and recognize gene expression clusters. Nucleic Acids Research.

[B10] Korbel J, Doerks T, Jensen L, Perez-Iratxeta C, Kaczanowski S, Hooper S, Andrade M, Bork P (2005). Systematic association of genes to phenotypes by genome and literature mining. PLoS Biology.

[B11] Blei D, Franks K, Jordan M, Mian I (2006). Statistical modeling of biomedical corpora: mining the *Caenorhabditis *Genetic Center Bibliography for genes related to aging. BMC Bioinformatics.

[B12] Raychaudhuri S, Schütze H, Altman R (2002). Using text analysis to identify functionally coherent gene groups. Genome Research.

[B13] Harris M, Clark J, Ireland A, Lomax J, Ashburner M, Foulger R, Eilbeck K, Lewis S, Marshall B, Mungall C, Richter J, Rubin G, Blake J, Bult C, Dolan M, Drabkin H, Eppig J, Hill D, Ni L, Ringwald M, Balakrishnan R, Cherry J, Christie K, Costanzo M, Dwight S, Engel S, Fisk D, Hirschman J, Hong E, Nash R, Sethuraman A, Theesfeld C, Botstein D, Dolinski K, Feierbach B, Berardini T, Mundodi S, Rhee S, Apweiler R, Barrell D, Camon E, Dimmer E, Lee V, Chisholm R, Gaudet P, Kibbe W, Kishore R, Schwarz E, Sternberg P, Gwinn M, Hannick L, Wortman J, Berriman M, Wood V, de la Cruz N, Tonellato P, Jaiswal P, Seigfried T, White R (2004). The Gene Ontology (GO) database and informatics resource. Nucleic Acids Research.

[B14] Zeeberg B, Feng W, Wang G, Wang M, Fojo A, Sunshine M, Narasimhan S, Kane D, Reinhold W, Lababidi S, Bussey K, Riss J, Barrett J, Weinstein J (2003). GoMiner: a resource for biological interpretation of genomic and proteomic data. Genome Biology.

[B15] GOTermFinder. http://www.yeastgenome.org.

[B16] Robinson M, Grigull J, Mohammad N, Hughes T (2002). FunSpec: a web-based cluster interpreter for yeast. BMC Bioinformatics.

[B17] Beißbarth T, Speed T (2004). GOstat: Find statistically overrepresented Gene Ontologies within a group of genes. Bioinformatics.

[B18] Berriz G, King O, Bryant B, Sander C, Roth F (2003). Characterizing gene sets with FuncAssociate. Bioinformatics.

[B19] Manning C, Schütze H (1999). Foundations of Statistical Natural Language Processing.

[B20] Salton G (1988). Automatic Text Processing: The Transformation Analysis and Retrieval of Information by Computer.

[B21] Moler E, Chow M, Mian I (2000). Analysis of molecular profile data using generative and discriminative methods. Physiological Genomics.

[B22] Moler E, Radisky D, Mian I (2000). Integrating naïve Bayes models and external knowledge to examine copper and iron homeostasis in *Saccharomyces cerevisiae*. Physiological Genomics.

[B23] Bhattacharjee A, Richards W, Staunton J, Li C, Monti S, Vasa P, Ladd C, Beheshti J, Bueno R, Gillette M, Loda M, Weber G, Mark E, Lander E, Wong W, Johnson B, Golub T, Sugarbaker D, Meyerson M (2001). Classification of human lung carcinomas by mRNA expression profiling reveals distinct adenocarcinoma subclasses. Proc Natl Acad Sci.

[B24] Monti S, Tamayo P, Mesirov J, Golub T (2003). Consensus clustering: a resampling-based method for class discovery and visualization of gene expression microarray data. Machine Learning.

[B25] Kellam P, Liu X, Martin N, Orengo C, Swift S, Tucker A (2001). Comparing, contrasting and combining clusters in viral gene expression data. Proceedings of the IDAMAP2001 Workshop 2001.

[B26] Jones C, Mackay A, Grigoriadis A, Cossu A, Reis-Filho J, Fulford L, Dexter T, Davies S, Bulmer K, Ford E, Parry S, Budroni M, Palmieri G, Neville A, O'Hare M, Lakhani S (2004). Expression profiling of purified normal human luminal and myoepithelial breast cells: identification of novel prognostic markers for breast cancer. Cancer Research.

[B27] Hofmann T, Puzicha J, Jordan M (1999). Learning from dyadic data. Advances in Neural Information Processing Systems.

[B28] Hoff P (2005). Model-based subspace clustering. Bayesian Analysis.

[B29] Hoff P (2005). Subset clustering of binary sequences, with an application to genomic abnormality data. Biometrics.

[B30] Glenisson P, Coessens B, van Vooren S, Mathys J, Moreau Y, de Moor B (2004). TXTGate: profiling gene groups with text-based information. Genome Biology.

[B31] Hurst L, Pál C, Lercher M (2004). The evolutionary dynamics of eukaryotic gene order. Nature Review Genetics.

[B32] Allinen M, Beroukhim R, Cai L, Brennan C, Lahti-Domenici J, Huang H, Porter D, Hu M, Chin L, Richardson A, Schnitt S, Sellers W, Polyak K (2004). Molecular characterization of the tumor microenvironment in breast cancer. Cancer Cell.

[B33] Jones C, Ford E, Gillett C, Ryder K, Merrett S, Reis-Filho J, Fulford L, Hanby A, Lakhani S (2004). Molecular cytogenetic identification of subgroups of grade III invasive ductal breast carcinomas with different clinical outcomes. Clinical Cancer Research.

[B34] Pruitt K, Maglott D (2001). RefSeq and LocusLink: NCBI gene-centered resources. Nucleic Acids Research.

[B35] RefSeq. http://www.ncbi.nlm.nih.gov/RefSeq.

[B36] Bioperl. http://www.bioperl.org.

[B37] GO. http://www.geneontology.org/.

[B38] CDD. http://www.ncbi.nlm.nih.gov/Structure/cdd/cdd.shtml.

[B39] Cheeseman P, Stutz J, Fayyad U, Piatetsky-Shapiro G, Smyth P, Uthurusamy R (1996). Bayesian Classification (AutoClass): Theory and Results. Advances in Knowledge Discovery and Data Mining.

[B40] Kuncheva L (2004). Combining Pattern Classifiers: Methods and Algorithms.

[B41] Fred A (2002). Finding consistent clusters in data partitions. Multiple Classifier Systems.

[B42] Strehl A, Ghosh J (2002). Cluster Ensembles – A Knowledge Reuse Framework for Combining Multiple Partitions. Journal of Machine Learning Research.

[B43] Topchy A, Jain A, Punch W (2004). A mixture model of clustering ensembles. Proceedings SIAM Conf on Data Mining.

[B44] C++ Boost Graph library. http://www.boost.org/libs/graph/doc/index.html.

[B45] KEGG. http://www.genome.jp/kegg.

[B46] UCSC Genome Browser. http://genome.ucsc.edu.

