# Supplementary material for: Ensemble attribute profile clustering: discovering and characterizing groups of genes with similar patterns of biological features
Source: BMC Bioinformatics. 2006 Mar 16;7:147. doi: 10.1186/1471-2105-7-147 (PMC1435935; doi:10.1186/1471-2105-7-147)
Supplement: Additional File 1 — Information on the 52 genes in the LUMINAL collection. For each gene, the "LocusID", "Symbol", "Name", "Cytoband Location", "GO Terms", "Domain Terms", "KEGG Pathway" and "OMIM" fields contain data taken from the July 2004 release of LocusLink. Genes are grouped by their assigned consensus clusters and ordered by their cytoband locations within each cluster. The GO and CDD terms shown are attributes in the gene attribute profiles used as input for probablistic clustering (explicit terms assigned to a gene by LocusLink plus implicit GO terms). The attributes marked with an asterisk are influential attributes, GO/CDD terms that occur with a frequency > 0.5 in clusters with two or more genes. [file 1471-2105-7-147-S1.html]

# Class 0 (24 LocusIDs)

| LocusLink | Symbol | Name | Cytoband Location | GO Terms | Domain Terms | KEGG Pathway | OMIM |
| --- | --- | --- | --- | --- | --- | --- | --- |
| 4582 | MUC1 | mucin 1, transmembrane | 1q21 | - [MF] GO:0003779: actin binding - [CC] GO:0005856: cytoskeleton - [CC] GO:0005887: integral to plasma membrane \* | - KOG2150: CCR4-NOT transcriptional regulation complex, NOT5 subunit [Transcription] - pfam01390: SEA domain |  |  |
| 10500 | SEMA6C | sema domain, transmembrane domain (TM), and cytoplasmic domain, (semaphorin) 6C | 1q21.2 | - [MF] GO:0004872: receptor activity \* - [BP] GO:0007275: development \* - [BP] GO:0007399: neurogenesis - [CC] GO:0016021: integral to membrane \* | - smart00423: domain found in Plexins, Semaphorins and Integrins - smart00630: semaphorin domain |  |  |
| 481 | ATP1B1 | ATPase, Na+/K+ transporting, beta 1 polypeptide | 1q24 | - [MF] GO:0005391: sodium/potassium-exchanging ATPase activity - [CC] GO:0005890: sodium/potassium-exchanging ATPase complex - [BP] GO:0006810: transport \* - [BP] GO:0006813: potassium ion transport - [BP] GO:0006814: sodium ion transport - [CC] GO:0016021: integral to membrane \* | - pfam00287: Sodium / potassium ATPase beta chain | - Oxidative phosphorylation |  |
| 9467 | SH3BP5 | SH3-domain binding protein 5 (BTK-associated) | 3p24.3 | - [MF] GO:0005070: SH3/SH2 adaptor protein activity - [CC] GO:0005737: cytoplasm \* - [BP] GO:0007165: signal transduction | - KOG2008: BTK-associated SH3-domain binding protein SAB [Signal transduction mechanisms] |  |  |
| 4820 | NKTR | natural killer-tumor recognition sequence | 3p23-p21 | - [MF] GO:0003755: peptidyl-prolyl cis-trans isomerase activity - [CC] GO:0005886: plasma membrane \* - [BP] GO:0006457: protein folding - [MF] GO:0016018: cyclosporin A binding - [MF] GO:0016853: isomerase activity | - KOG0546: HSP90 co-chaperone CPR7/Cyclophilin [Posttranslational modification, protein turnover, chaperones] - KOG2002: TPR-containing nuclear phosphoprotein that regulates K(+) uptake [Inorganic ion transport and metabolism] - pfam00160: Cyclophilin type peptidyl-prolyl cis-trans isomerase |  |  |
| 7869 | SEMA3B | sema domain, immunoglobulin domain (Ig), short basic domain, secreted, (semaphorin) 3B | 3p21.3 | - [MF] GO:0004872: receptor activity \* - [CC] GO:0005783: endoplasmic reticulum \* - [BP] GO:0007267: cell-cell signaling - [BP] GO:0007275: development \* - [BP] GO:0007411: axon guidance - [CC] GO:0016020: membrane \* | - KOG3611: Semaphorins [Signal transduction mechanisms] |  |  |
| 5918 | RARRES1 | retinoic acid receptor responder (tazarotene induced) 1 | 3q25.32 | - [BP] GO:0008285: negative regulation of cell proliferation - [CC] GO:0016021: integral to membrane \* |  |  |  |
| 4241 | MFI2 | antigen p97 (melanoma associated) identified by monoclonal antibodies 133.2 and 96.5 | 3q28-q29 | - [CC] GO:0005576: extracellular \* - [CC] GO:0005887: integral to plasma membrane \* - [BP] GO:0006810: transport \* - [BP] GO:0006826: iron ion transport - [BP] GO:0006879: iron ion homeostasis - [MF] GO:0008199: ferric iron binding - [CC] GO:0016020: membrane \* | - pfam00405: Transferrin |  |  |
| 934 | CD24 | CD24 antigen (small cell lung carcinoma cluster 4 antigen) | 6q21 | - [CC] GO:0005886: plasma membrane \* - [BP] GO:0006959: humoral immune response |  |  |  |
| 1364 | CLDN4 | claudin 4 | 7q11.23 | - [MF] GO:0004888: transmembrane receptor activity - [MF] GO:0005198: structural molecule activity \* - [CC] GO:0005887: integral to plasma membrane \* - [CC] GO:0005923: tight junction - [BP] GO:0009405: pathogenesis | - pfam00822: PMP-22/EMP/MP20/Claudin family |  |  |
| 3934 | LCN2 | lipocalin 2 (oncogene 24p3) | 9q34 | - [MF] GO:0005215: transporter activity \* - [MF] GO:0005488: binding \* - [CC] GO:0005625: soluble fraction - [CC] GO:0005737: cytoplasm \* - [BP] GO:0006810: transport \* | - pfam00061: Lipocalin / cytosolic fatty-acid binding protein family |  |  |
| 311 | ANXA11 | annexin A11 | 10q23 | - [MF] GO:0005509: calcium ion binding - [MF] GO:0005515: protein binding \* - [MF] GO:0005544: calcium-dependent phospholipid binding - [CC] GO:0005635: nuclear membrane - [CC] GO:0005654: nucleoplasm - [CC] GO:0005737: cytoplasm \* - [BP] GO:0006955: immune response \* | - KOG0819: Annexin [Intracellular trafficking, secretion, and vesicular transport] |  |  |
| 977 | CD151 | CD151 antigen | 11p15.5 | - [MF] GO:0005515: protein binding \* - [CC] GO:0005624: membrane fraction - [CC] GO:0005887: integral to plasma membrane \* - [BP] GO:0007155: cell adhesion \* | - KOG3882: Tetraspanin family integral membrane protein [General function prediction only] |  |  |
| 2065 | ERBB3 | v-erb-b2 erythroblastic leukemia viral oncogene homolog 3 (avian) | 12q13 | - [MF] GO:0004872: receptor activity \* - [MF] GO:0005006: epidermal growth factor receptor activity - [MF] GO:0005524: ATP binding - [CC] GO:0005887: integral to plasma membrane \* - [BP] GO:0006468: protein amino acid phosphorylation - [BP] GO:0007169: transmembrane receptor protein tyrosine kinase signaling pathway - [MF] GO:0016740: transferase activity | - cd00064: Furin-like repeats - cd00192: Tyrosine kinase, catalytic domain - KOG1025: Epidermal growth factor receptor EGFR and related tyrosine kinases [Signal transduction mechanisms] - pfam00757: Furin-like cysteine rich region - pfam01030: Receptor L domain |  |  |
| 5720 | PSME1 | proteasome (prosome, macropain) activator subunit 1 (PA28 alpha) | 14q11.2 | - [CC] GO:0000502: proteasome complex (sensu Eukarya) - [BP] GO:0006955: immune response \* - [CC] GO:0008537: proteasome activator complex - [MF] GO:0008538: proteasome activator activity | - KOG4470: Proteasome activator subunit [Posttranslational modification, protein turnover, chaperones] |  |  |
| 3958 | LGALS3 | lectin, galactoside-binding, soluble, 3 (galectin 3) | 14q21-q22 | - [MF] GO:0005529: sugar binding - [CC] GO:0005576: extracellular \* - [CC] GO:0005634: nucleus \* - [CC] GO:0005886: plasma membrane \* - [BP] GO:0007157: heterophilic cell adhesion - [MF] GO:0019863: IgE binding | - cd00070: Galectin/galactose-binding lectin |  |  |
| 5652 | PRSS8 | protease, serine, 8 (prostasin) | 16p11.2 | - [MF] GO:0004263: chymotrypsin activity - [MF] GO:0004295: trypsin activity - [CC] GO:0005615: extracellular space - [CC] GO:0005886: plasma membrane \* - [BP] GO:0006508: proteolysis and peptidolysis \* - [CC] GO:0016021: integral to membrane \* - [MF] GO:0016787: hydrolase activity \* | - cd00190: Trypsin-like serine protease |  |  |
| 6376 | CX3CL1 | chemokine (C-X3-C motif) ligand 1 | 16q13 | - [MF] GO:0005515: protein binding \* - [CC] GO:0005576: extracellular \* - [BP] GO:0006955: immune response \* - [BP] GO:0007155: cell adhesion \* - [MF] GO:0008009: chemokine activity - [CC] GO:0009986: cell surface - [CC] GO:0016021: integral to membrane \* - [BP] GO:0016338: calcium-independent cell-cell adhesion - [BP] GO:0019221: cytokine and chemokine mediated signaling pathway - [BP] GO:0030595: immune cell chemotaxis - [BP] GO:0050729: positive regulation of inflammatory response - [BP] GO:0050902: leukocyte adhesive activation | - cd00274: Small secreted cytokine (intecrine/chemokine) domain subgroup CXXXC/fractaline/neurotactin | - Cytokine-cytokine receptor interaction |  |
| 9368 | SLC9A3R1 | solute carrier family 9 (sodium/hydrogen exchanger), isoform 3 regulator 1 | 17q25.2 | - [MF] GO:0005515: protein binding \* - [BP] GO:0006461: protein complex assembly - [CC] GO:0015629: actin cytoskeleton - [BP] GO:0016055: Wnt receptor signaling pathway | - cd00992: PDZ domain found in a variety of Eumetazoan signaling molecules, often in tandem arrangements |  |  |
| 4616 | GADD45B | growth arrest and DNA-damage-inducible, beta | 19p13.3 | - [BP] GO:0000185: activation of MAPKKK - [MF] GO:0003735: structural constituent of ribosome - [CC] GO:0005840: ribosome - [BP] GO:0006412: protein biosynthesis - [BP] GO:0006915: apoptosis - [BP] GO:0006950: response to stress - [BP] GO:0030154: cell differentiation | - pfam01248: Ribosomal protein L7Ae/L30e/S12e/Gadd45 family | - MAPK signaling pathway - Cell cycle |  |
| 8612 | PPAP2C | phosphatidic acid phosphatase type 2C | 19p13 | - [MF] GO:0004721: phosphoprotein phosphatase activity - [MF] GO:0008195: phosphatidate phosphatase activity - [CC] GO:0016021: integral to membrane \* - [MF] GO:0016787: hydrolase activity \* | - KOG3030: Lipid phosphate phosphatase and related enzymes of the PAP2 family [Lipid transport and metabolism] | - Glycerolipid metabolism - Phospholipid degradation - Glycosphingolipid metabolism |  |
| 5653 | KLK6 | kallikrein 6 (neurosin, zyme) | 19q13.3 | - [MF] GO:0004263: chymotrypsin activity - [MF] GO:0004293: tissue kallikrein activity - [MF] GO:0004295: trypsin activity - [MF] GO:0005515: protein binding \* - [CC] GO:0005576: extracellular \* - [CC] GO:0005737: cytoplasm \* - [BP] GO:0007417: central nervous system development - [BP] GO:0009611: response to wounding - [BP] GO:0016540: protein autoprocessing - [MF] GO:0016787: hydrolase activity \* - [BP] GO:0030574: collagen catabolism - [BP] GO:0042246: tissue regeneration - [BP] GO:0042445: hormone metabolism - [BP] GO:0042552: myelination - [BP] GO:0042982: amyloid precursor protein metabolism - [BP] GO:0045595: regulation of cell differentiation | - cd00190: Trypsin-like serine protease |  |  |
| 25825 | BACE2 | beta-site APP-cleaving enzyme 2 | 21q22.3 | - [MF] GO:0004194: pepsin A activity - [CC] GO:0005624: membrane fraction - [BP] GO:0006464: protein modification - [BP] GO:0006509: membrane protein ectodomain proteolysis - [BP] GO:0006509: membrane protein ectodomain proteolysis - [MF] GO:0008233: peptidase activity - [MF] GO:0009049: aspartic-type signal peptidase activity - [BP] GO:0009306: protein secretion - [CC] GO:0016021: integral to membrane \* - [CC] GO:0016021: integral to membrane \* - [BP] GO:0016486: peptide hormone processing - [BP] GO:0016486: peptide hormone processing - [MF] GO:0016787: hydrolase activity \* - [BP] GO:0042985: negative regulation of amyloid precursor protein biosynthesis - [BP] GO:0042985: negative regulation of amyloid precursor protein biosynthesis | - KOG1339: Aspartyl protease [Posttranslational modification, protein turnover, chaperones] |  |  |
| 23654 | PLXNB2 | plexin B2 | 22q13.33 | - [MF] GO:0004872: receptor activity \* - [BP] GO:0007275: development \* - [CC] GO:0016020: membrane \* |  |  |  |

# Class 1 (20 LocusIDs)

| LocusLink | Symbol | Name | Cytoband Location | GO Terms | Domain Terms | KEGG Pathway | OMIM |
| --- | --- | --- | --- | --- | --- | --- | --- |
| 5768 | QSCN6 | quiescin Q6 | 1q24 | - [BP] GO:0000074: regulation of cell cycle - [BP] GO:0001558: regulation of cell growth - [MF] GO:0005489: electron transporter activity - [BP] GO:0006118: electron transport \* - [BP] GO:0008151: cell growth and/or maintenance \* - [BP] GO:0008285: negative regulation of cell proliferation - [CC] GO:0008372: cellular\_component unknown | - KOG1731: FAD-dependent sulfhydryl oxidase/quiescin and related proteins [Cell cycle control, cell division, chromosome partitioning] - pfam00085: Thioredoxin - pfam04777: Erv1 / Alr family |  |  |
| 57730 | KIAA1641 | KIAA1641 protein | 2q11.2 |  |  |  |  |
| 10550 | JWA | cytoskeleton related vitamin A responsive protein | 3p14 |  | - KOG4050: Glutamate transporter EAAC1-interacting protein GTRAP3-18 [Amino acid transport and metabolism, Signal transduction mechanisms] |  |  |
| 11343 | MGLL | monoglyceride lipase | 3q21.3 | - [MF] GO:0004622: lysophospholipase activity - [MF] GO:0004759: serine esterase activity - [BP] GO:0006629: lipid metabolism - [BP] GO:0006725: aromatic compound metabolism - [BP] GO:0006954: inflammatory response - [MF] GO:0016787: hydrolase activity - [MF] GO:0047372: acylglycerol lipase activity | - COG2267: Lysophospholipase [Lipid metabolism] - KOG1455: Lysophospholipase [Lipid transport and metabolism] |  |  |
| 2745 | GLRX | glutaredoxin (thioltransferase) | 5q14 | - [MF] GO:0005489: electron transporter activity - [BP] GO:0006118: electron transport \* - [MF] GO:0015038: glutathione disulfide oxidoreductase activity | - KOG1752: Glutaredoxin and related proteins [Posttranslational modification, protein turnover, chaperones] |  |  |
| 8935 | SCAP2 | src family associated phosphoprotein 2 | 7p21-p15 | - [MF] GO:0005070: SH3/SH2 adaptor protein activity - [BP] GO:0006461: protein complex assembly - [BP] GO:0007165: signal transduction - [MF] GO:0016301: kinase activity | - smart00233: Pleckstrin homology domain - smart00326: Src homology 3 domains |  |  |
| 10659 | CUGBP2 | CUG triplet repeat, RNA binding protein 2 | 10p13 | - [MF] GO:0003723: RNA binding - [MF] GO:0003723: RNA binding - [BP] GO:0006396: RNA processing - [BP] GO:0007528: neuromuscular junction development - [BP] GO:0008016: regulation of heart rate | - KOG0144: RNA-binding protein CUGBP1/BRUNO (RRM superfamily) [RNA processing and modification] - smart00360: RNA recognition motif |  |  |
| 5092 | PCBD | 6-pyruvoyl-tetrahydropterin synthase/dimerization cofactor of hepatocyte nuclear factor 1 alpha (TCF1) | 10q22 |  | - pfam01329: Pterin 4 alpha carbinolamine dehydratase |  | - 264070 |
| 1474 | CST6 | cystatin E/M | 11q13 | - [MF] GO:0004869: cysteine protease inhibitor activity - [BP] GO:0009653: morphogenesis | - smart00043: Cystatin-like domain |  |  |
| 11145 | HRASLS3 | HRAS-like suppressor 3 | 11q13.1 | - [BP] GO:0000004: biological\_process unknown - [MF] GO:0005554: molecular\_function unknown - [CC] GO:0008372: cellular\_component unknown | - pfam04970: NC domain |  |  |
| 5920 | RARRES3 | retinoic acid receptor responder (tazarotene induced) 3 | 11q23 |  | - pfam04970: NC domain |  |  |
| 7127 | TNFAIP2 | tumor necrosis factor, alpha-induced protein 2 | 14q32 | - [BP] GO:0001525: angiogenesis - [CC] GO:0005615: extracellular space \* | - KOG2286: Exocyst complex subunit SEC6 [Intracellular trafficking, secretion, and vesicular transport] |  |  |
| 9235 | NK4 | natural killer cell transcript 4 | 16p13.3 | - [CC] GO:0005615: extracellular space \* - [BP] GO:0006955: immune response \* - [BP] GO:0007155: cell adhesion \* |  |  |  |
| 283846 | KIAA0220 | PI-3-kinase-related kinase SMG-1-like | 16p12.3 | - [BP] GO:0000004: biological\_process unknown - [MF] GO:0005554: molecular\_function unknown - [CC] GO:0008372: cellular\_component unknown |  |  |  |
| 3965 | LGALS9 | lectin, galactoside-binding, soluble, 9 (galectin 9) | 17q11.2 | - [MF] GO:0004871: signal transducer activity \* - [MF] GO:0005529: sugar binding - [MF] GO:0005534: galactose binding - [BP] GO:0007157: heterophilic cell adhesion - [BP] GO:0043123: positive regulation of I-kappaB kinase/NF-kappaB cascade | - cd00070: Galectin/galactose-binding lectin |  |  |
| 79170 | MGC11242 | hypothetical protein MGC11242 | 17q21.32 |  |  |  |  |
| 57153 | CTL2 | CTL2 gene | 19p13.1 | - [MF] GO:0004871: signal transducer activity \* - [BP] GO:0043123: positive regulation of I-kappaB kinase/NF-kappaB cascade | - KOG1362: Choline transporter-like protein [Lipid transport and metabolism] |  |  |
| 10272 | FSTL3 | follistatin-like 3 (secreted glycoprotein) | 19p13 | - [CC] GO:0005615: extracellular space \* - [BP] GO:0008151: cell growth and/or maintenance \* | - cd00104: Kazal type serine protease inhibitors and follistatin-like domains - KOG4004: Matricellular protein Osteonectin/SPARC/BM-40 [Extracellular structures] |  |  |
| 10406 | WFDC2 | WAP four-disulfide core domain 2 | 20q12-q13.2 | - [MF] GO:0004867: serine-type endopeptidase inhibitor activity - [CC] GO:0005615: extracellular space \* - [BP] GO:0006508: proteolysis and peptidolysis - [BP] GO:0007283: spermatogenesis | - cd00199: whey acidic protein-type four-disulfide core domains - KOG4802: Adhesion-type protein [Extracellular structures] - pfam00095: WAP-type (Whey Acidic Protein) 'four-disulfide core' - smart00217: Four-disulfide core domains |  |  |
| 8785 | MATN4 | matrilin 4 | 20q13.1-q13.2 | - [MF] GO:0005201: extracellular matrix structural constituent - [MF] GO:0005518: collagen binding - [CC] GO:0005578: extracellular matrix - [BP] GO:0030198: extracellular matrix organization and biogenesis | - KOG1214: Nidogen and related basement membrane protein proteins [Cell wall/membrane/envelope biogenesis, Extracellular structures] - KOG1215: Low-density lipoprotein receptors containing Ca2+-binding EGF-like domains [Signal transduction mechanisms] - KOG1217: Fibrillins and related proteins containing Ca2+-binding EGF-like domains [Signal transduction mechanisms] - pfam00092: von Willebrand factor type A domain |  |  |

# Class 2 (4 LocusIDs)

| LocusLink | Symbol | Name | Cytoband Location | GO Terms | Domain Terms | KEGG Pathway | OMIM |
| --- | --- | --- | --- | --- | --- | --- | --- |
| 53335 | BCL11A | B-cell CLL/lymphoma 11A (zinc finger protein) | 2p16.1 | - [MF] GO:0003676: nucleic acid binding \* - [CC] GO:0005634: nucleus \* - [CC] GO:0005737: cytoplasm - [BP] GO:0006355: regulation of transcription, DNA-dependent \* - [MF] GO:0008270: zinc ion binding - [BP] GO:0030097: hemopoiesis | - KOG1074: Transcriptional repressor SALM [Transcription] - KOG2462: C2H2-type Zn-finger protein [Transcription] |  |  |
| 2971 | GTF3A | general transcription factor IIIA | 13q12.3-q13.1 | - [MF] GO:0003677: DNA binding \* - [MF] GO:0003709: RNA polymerase III transcription factor activity - [MF] GO:0003723: RNA binding - [CC] GO:0005634: nucleus \* - [BP] GO:0006355: regulation of transcription, DNA-dependent \* - [BP] GO:0006383: transcription from Pol III promoter - [MF] GO:0008270: zinc ion binding - [BP] GO:0009303: rRNA transcription | - COG5048: FOG: Zn-finger [General function prediction only] - KOG1074: Transcriptional repressor SALM [Transcription] - KOG3608: Zn finger proteins [General function prediction only] |  |  |
| 677 | ZFP36L1 | zinc finger protein 36, C3H type-like 1 | 14q22-q24 | - [MF] GO:0003700: transcription factor activity - [CC] GO:0005634: nucleus \* | - KOG1677: CCCH-type Zn-finger protein [General function prediction only] - pfam00642: Zinc finger C-x8-C-x5-C-x3-H type (and similar) - pfam04553: Tis11B like protein, C terminus |  |  |
| 29855 | UBN1 | ubinuclein 1 | 16p13.3 | - [MF] GO:0003700: transcription factor activity - [CC] GO:0005634: nucleus \* - [BP] GO:0006357: regulation of transcription from Pol II promoter | - KOG4786: Ubinuclein, nuclear protein interacting with cellular and viral transcription factors [Transcription, Signal transduction mechanisms] |  |  |

# Class 3 (4 LocusIDs)

| LocusLink | Symbol | Name | Cytoband Location | GO Terms | Domain Terms | KEGG Pathway | OMIM |
| --- | --- | --- | --- | --- | --- | --- | --- |
| 1718 | DHCR24 | 24-dehydrocholesterol reductase | 1p33-p31.1 | - [CC] GO:0005783: endoplasmic reticulum - [CC] GO:0005794: Golgi apparatus - [BP] GO:0006118: electron transport \* - [BP] GO:0006695: cholesterol biosynthesis - [CC] GO:0016021: integral to membrane \* - [MF] GO:0016491: oxidoreductase activity \* | - KOG1262: FAD-binding protein DIMINUTO [General function prediction only] - pfam01565: FAD binding domain | - Fatty acid biosynthesis (path 2) - 1,1,1-Trichloro-2,2-bis(4-chlorophenyl)ethane (DDT) degradation - Tryptophan metabolism - Propanoate metabolism | - 602398 |
| 4508 | MTATP6 | ATP synthase 6 | mitochondrion | - [CC] GO:0000276: proton-transporting ATP synthase complex, coupling factor F(o) (sensu Eukarya) - [CC] GO:0005739: mitochondrion \* - [MF] GO:0015078: hydrogen ion transporter activity \* - [BP] GO:0015986: ATP synthesis coupled proton transport - [BP] GO:0015992: proton transport - [CC] GO:0016469: proton-transporting two-sector ATPase complex - [MF] GO:0016820: hydrolase activity, acting on acid anhydrides, catalyzing transmembrane movement of substances |  | - Oxidative phosphorylation - ATP synthesis - Photosynthesis |  |
| 4513 | MTCO2 | cytochrome c oxidase II | mitochondrion | - [MF] GO:0004129: cytochrome-c oxidase activity - [MF] GO:0005507: copper ion binding - [CC] GO:0005739: mitochondrion \* - [CC] GO:0005746: mitochondrial electron transport chain - [BP] GO:0006118: electron transport \* - [BP] GO:0006123: mitochondrial electron transport, cytochrome c to oxygen - [CC] GO:0016021: integral to membrane \* - [MF] GO:0016491: oxidoreductase activity \* - [CC] GO:0019866: inner membrane |  | - Oxidative phosphorylation - Electron Transport System, Complex IV |  |
| 4538 | MTND4 | NADH dehydrogenase 4 | mitochondrion | - [CC] GO:0005739: mitochondrion \* - [CC] GO:0005747: respiratory chain complex I (sensu Eukarya) - [BP] GO:0006120: mitochondrial electron transport, NADH to ubiquinone - [MF] GO:0008137: NADH dehydrogenase (ubiquinone) activity - [MF] GO:0008137: NADH dehydrogenase (ubiquinone) activity - [MF] GO:0016491: oxidoreductase activity \* - [BP] GO:0042773: ATP synthesis coupled electron transport |  | - Ubiquinone biosynthesis - Oxidative phosphorylation |  |
