# Supplementary material for: Ensemble attribute profile clustering: discovering and characterizing groups of genes with similar patterns of biological features
Source: BMC Bioinformatics. 2006 Mar 16;7:147. doi: 10.1186/1471-2105-7-147 (PMC1435935; doi:10.1186/1471-2105-7-147)
Supplement: Additional File 2 — Information on the GO and CDD attributes associated with the consensus clusters discovered for genes in the LUMINAL collection. For each consensus cluster, the file shows the number of genes assigned to the cluster, along with the absolute count and relative frequency of all attributes associated with the cluster's genes. The section labeled "Summary" shows all the attributes associated with genes in the data set, ordered by average count across all consensus clusters. [file 1471-2105-7-147-S2.html]

Attribute breakdown

# Class 0 (24 LocusIDs)

| ID | Name | Count | Relative frequency |
| --- | --- | --- | --- |
| GO:0005623 | [CC] cell | 24 | 1.00 |
| GO:0016020 | [CC] membrane | 18 | 0.75 |
| GO:0016021 | [CC] integral to membrane | 12 | 0.50 |
| GO:0005488 | [MF] binding | 11 | 0.46 |
| GO:0005886 | [CC] plasma membrane | 10 | 0.42 |
| GO:0005622 | [CC] intracellular | 10 | 0.42 |
| GO:0005737 | [CC] cytoplasm | 9 | 0.38 |
| GO:0007154 | [BP] cell communication | 8 | 0.33 |
| GO:0050875 | [BP] cellular physiological process | 7 | 0.29 |
| GO:0019538 | [BP] protein metabolism | 7 | 0.29 |
| GO:0003824 | [MF] catalytic activity | 7 | 0.29 |
| GO:0008152 | [BP] metabolism | 7 | 0.29 |
| GO:0004871 | [MF] signal transducer activity | 7 | 0.29 |
| GO:0005515 | [MF] protein binding | 7 | 0.29 |
| GO:0005887 | [CC] integral to plasma membrane | 6 | 0.25 |
| GO:0004872 | [MF] receptor activity | 5 | 0.21 |
| GO:0005576 | [CC] extracellular | 5 | 0.21 |
| GO:0007275 | [BP] development | 5 | 0.21 |
| GO:0016787 | [MF] hydrolase activity | 5 | 0.21 |
| GO:0006955 | [BP] immune response | 4 | 0.17 |
| GO:0006952 | [BP] defense response | 4 | 0.17 |
| GO:0050874 | [BP] organismal physiological process | 4 | 0.17 |
| GO:0008151 | [BP] cell growth and/or maintenance | 4 | 0.17 |
| GO:0005634 | [CC] nucleus | 3 | 0.12 |
| GO:0006810 | [BP] transport | 3 | 0.12 |
| GO:0006508 | [BP] proteolysis and peptidolysis | 3 | 0.12 |
| GO:0007155 | [BP] cell adhesion | 3 | 0.12 |
| GO:0000267 | [CC] cell fraction | 3 | 0.12 |
| GO:0005198 | [MF] structural molecule activity | 2 | 0.08 |
| GO:0005215 | [MF] transporter activity | 2 | 0.08 |
| GO:0016337 | [BP] cell-cell adhesion | 2 | 0.08 |
| GO:0005783 | [CC] endoplasmic reticulum | 2 | 0.08 |

# Class 1 (20 LocusIDs)

| ID | Name | Count | Relative frequency |
| --- | --- | --- | --- |
| GO:0008152 | [BP] metabolism | 6 | 0.30 |
| GO:0005576 | [CC] extracellular | 5 | 0.25 |
| GO:0007275 | [BP] development | 5 | 0.25 |
| GO:0007154 | [BP] cell communication | 4 | 0.20 |
| GO:0005615 | [CC] extracellular space | 4 | 0.20 |
| GO:0050874 | [BP] organismal physiological process | 3 | 0.15 |
| GO:0050875 | [BP] cellular physiological process | 3 | 0.15 |
| GO:0005488 | [MF] binding | 3 | 0.15 |
| GO:0003824 | [MF] catalytic activity | 3 | 0.15 |
| GO:0004871 | [MF] signal transducer activity | 3 | 0.15 |
| GO:0006955 | [BP] immune response | 2 | 0.10 |
| GO:0006952 | [BP] defense response | 2 | 0.10 |
| GO:0005215 | [MF] transporter activity | 2 | 0.10 |
| GO:0019538 | [BP] protein metabolism | 2 | 0.10 |
| GO:0007155 | [BP] cell adhesion | 2 | 0.10 |
| GO:0008151 | [BP] cell growth and/or maintenance | 2 | 0.10 |
| GO:0006118 | [BP] electron transport | 2 | 0.10 |

# Class 2 (4 LocusIDs)

| ID | Name | Count | Relative frequency |
| --- | --- | --- | --- |
| GO:0003676 | [MF] nucleic acid binding | 4 | 1.00 |
| GO:0005634 | [CC] nucleus | 4 | 1.00 |
| GO:0005488 | [MF] binding | 4 | 1.00 |
| GO:0005622 | [CC] intracellular | 4 | 1.00 |
| GO:0005623 | [CC] cell | 4 | 1.00 |
| GO:0030528 | [MF] transcription regulator activity | 3 | 0.75 |
| GO:0006355 | [BP] regulation of transcription, DNA-dependent | 3 | 0.75 |
| GO:0003677 | [MF] DNA binding | 3 | 0.75 |
| GO:0008152 | [BP] metabolism | 3 | 0.75 |
| GO:0046914 | [MF] transition metal ion binding | 2 | 0.50 |

# Class 3 (4 LocusIDs)

| ID | Name | Count | Relative frequency |
| --- | --- | --- | --- |
| GO:0003824 | [MF] catalytic activity | 4 | 1.00 |
| GO:0005622 | [CC] intracellular | 4 | 1.00 |
| GO:0008152 | [BP] metabolism | 4 | 1.00 |
| GO:0005623 | [CC] cell | 4 | 1.00 |
| GO:0005737 | [CC] cytoplasm | 4 | 1.00 |
| GO:0016020 | [CC] membrane | 4 | 1.00 |
| GO:0016491 | [MF] oxidoreductase activity | 3 | 0.75 |
| GO:0005739 | [CC] mitochondrion | 3 | 0.75 |
| GO:0016021 | [CC] integral to membrane | 3 | 0.75 |
| GO:0005215 | [MF] transporter activity | 3 | 0.75 |
| GO:0015078 | [MF] hydrogen ion transporter activity | 3 | 0.75 |
| GO:0006118 | [BP] electron transport | 3 | 0.75 |

# Summary

| ID | Name | 0 | 1 | 2 | 3 |
| --- | --- | --- | --- | --- | --- |
| GO:0008152 | metabolism | 7 | 6 | 3 | 4 |
| GO:0005488 | binding | 11 | 3 | 4 | 1 |
| GO:0005623 | cell | 24 | 0 | 4 | 4 |
| GO:0005622 | intracellular | 10 | 0 | 4 | 4 |
| GO:0005737 | cytoplasm | 9 | 0 | 1 | 4 |
| GO:0003824 | catalytic activity | 7 | 3 | 0 | 4 |
| GO:0050875 | cellular physiological process | 7 | 3 | 0 | 1 |
| GO:0007275 | development | 5 | 5 | 1 | 0 |
| GO:0016787 | hydrolase activity | 5 | 1 | 0 | 1 |
| GO:0008151 | cell growth and/or maintenance | 4 | 2 | 0 | 1 |
| GO:0005215 | transporter activity | 2 | 2 | 0 | 3 |
| GO:0046914 | transition metal ion binding | 1 | 0 | 2 | 1 |
| GO:0016020 | membrane | 18 | 0 | 0 | 4 |
| GO:0016021 | integral to membrane | 12 | 0 | 0 | 3 |
| GO:0007154 | cell communication | 8 | 4 | 0 | 0 |
| GO:0004871 | signal transducer activity | 7 | 3 | 0 | 0 |
| GO:0005576 | extracellular | 5 | 5 | 0 | 0 |
| GO:0019538 | protein metabolism | 7 | 2 | 0 | 0 |
| GO:0005515 | protein binding | 7 | 1 | 0 | 0 |
| GO:0050874 | organismal physiological process | 4 | 3 | 0 | 0 |
| GO:0005634 | nucleus | 3 | 0 | 4 | 0 |
| GO:0006952 | defense response | 4 | 2 | 0 | 0 |
| GO:0006955 | immune response | 4 | 2 | 0 | 0 |
| GO:0007155 | cell adhesion | 3 | 2 | 0 | 0 |
| GO:0005615 | extracellular space | 1 | 4 | 0 | 0 |
| GO:0006118 | electron transport | 0 | 2 | 0 | 3 |
| GO:0003676 | nucleic acid binding | 0 | 1 | 4 | 0 |
| GO:0006508 | proteolysis and peptidolysis | 3 | 1 | 0 | 0 |
| GO:0006810 | transport | 3 | 0 | 0 | 1 |
| GO:0016491 | oxidoreductase activity | 0 | 1 | 0 | 3 |
| GO:0016337 | cell-cell adhesion | 2 | 1 | 0 | 0 |
| GO:0005198 | structural molecule activity | 2 | 1 | 0 | 0 |
| GO:0005783 | endoplasmic reticulum | 2 | 0 | 0 | 1 |
| GO:0005886 | plasma membrane | 10 | 0 | 0 | 0 |
| GO:0005887 | integral to plasma membrane | 6 | 0 | 0 | 0 |
| GO:0004872 | receptor activity | 5 | 0 | 0 | 0 |
| GO:0000267 | cell fraction | 3 | 0 | 0 | 0 |
| GO:0003677 | DNA binding | 0 | 0 | 3 | 0 |
| GO:0006355 | regulation of transcription, DNA-dependent | 0 | 0 | 3 | 0 |
| GO:0030528 | transcription regulator activity | 0 | 0 | 3 | 0 |
| GO:0015078 | hydrogen ion transporter activity | 0 | 0 | 0 | 3 |
| GO:0005739 | mitochondrion | 0 | 0 | 0 | 3 |
