# Supplementary material for: Ensemble attribute profile clustering: discovering and characterizing groups of genes with similar patterns of biological features
Source: BMC Bioinformatics. 2006 Mar 16;7:147. doi: 10.1186/1471-2105-7-147 (PMC1435935; doi:10.1186/1471-2105-7-147)
Supplement: Additional File 3 — Information on the 89 genes in the consensus clusters discovered and characterized for the MYOEPITHELIAL collection, along with their associated GO and CDD attributes. The formats are the same as for luminal-all.html and luminal-attrfreq.html. [file 1471-2105-7-147-S3.html]

# Class 0 (48 LocusIDs)

| LocusLink | Symbol | Name | Cytoband Location | GO Terms | Domain Terms | KEGG Pathway | OMIM |
| --- | --- | --- | --- | --- | --- | --- | --- |
| 3399 | ID3 | inhibitor of DNA binding 3, dominant negative helix-loop-helix protein | 1p36.13-p36.12 | - [MF] GO:0003714: transcription corepressor activity - [CC] GO:0005634: nucleus \* - [BP] GO:0007275: development \* | - cd00083: Helix-loop-helix domain, found in specific DNA- binding proteins that act as transcription factors | - TGF-beta signaling pathway |  |
| 5052 | PRDX1 | peroxiredoxin 1 | 1p34.1 | - [BP] GO:0001501: skeletal development \* - [MF] GO:0004601: peroxidase activity - [BP] GO:0008283: cell proliferation \* - [MF] GO:0016491: oxidoreductase activity \* | - KOG0852: Alkyl hydroperoxide reductase, thiol specific antioxidant and related enzymes [Posttranslational modification, protein turnover, chaperones] |  |  |
| 6273 | S100A2 | S100 calcium binding protein A2 | 1q21 | - [BP] GO:0000004: biological\_process unknown - [MF] GO:0005509: calcium ion binding \* - [CC] GO:0008372: cellular\_component unknown | - cd00213: S-100/ICaBP-like domain |  |  |
| 6278 | S100A7 | S100 calcium binding protein A7 (psoriasin 1) | 1q21 | - [MF] GO:0005509: calcium ion binding \* - [BP] GO:0008544: epidermis development \* | - cd00213: S-100/ICaBP-like domain |  |  |
| 6699 | SPRR1B | small proline-rich protein 1B (cornifin) | 1q21-q22 | - [MF] GO:0005198: structural molecule activity \* - [CC] GO:0005737: cytoplasm \* - [CC] GO:0005882: intermediate filament - [BP] GO:0008544: epidermis development \* | - pfam02389: Cornifin (SPRR) family |  |  |
| 3398 | ID2 | inhibitor of DNA binding 2, dominant negative helix-loop-helix protein | 2p25 | - [CC] GO:0005634: nucleus \* - [BP] GO:0007275: development \* | - cd00083: Helix-loop-helix domain, found in specific DNA- binding proteins that act as transcription factors | - TGF-beta signaling pathway |  |
| 10797 | MTHFD2 | methylene tetrahydrofolate dehydrogenase (NAD+ dependent), methenyltetrahydrofolate cyclohydrolase | 2p13.1 | - [MF] GO:0000287: magnesium ion binding \* - [MF] GO:0004477: methenyltetrahydrofolate cyclohydrolase activity - [MF] GO:0004487: methylenetetrahydrofolate dehydrogenase (NAD+) activity - [MF] GO:0005489: electron transporter activity \* - [CC] GO:0005739: mitochondrion \* - [BP] GO:0006730: one-carbon compound metabolism - [BP] GO:0009396: folic acid and derivative biosynthesis - [MF] GO:0016491: oxidoreductase activity \* - [MF] GO:0016787: hydrolase activity \* | - KOG0089: Methylenetetrahydrofolate dehydrogenase/methylenetetrahydrofolate cyclohydrolase [Coenzyme transport and metabolism] | - Glyoxylate and dicarboxylate metabolism - One carbon pool by folate |  |
| 1290 | COL5A2 | collagen, type V, alpha 2 | 2q14-q32 | - [MF] GO:0005201: extracellular matrix structural constituent - [CC] GO:0005581: collagen - [CC] GO:0005588: collagen type V - [CC] GO:0005737: cytoplasm \* - [BP] GO:0006817: phosphate transport - [BP] GO:0008151: cell growth and/or maintenance \* | - KOG3546: Collagens (type XV) [Extracellular structures] - pfam00093: von Willebrand factor type C domain - smart00038: Fibrillar collagens C-terminal domain |  | - 130000 |
| 55627 | FLJ20297 | hypothetical protein FLJ20297 | 2q21.2 |  | - KOG4396: Predicted membrane-anchored protein [Function unknown] |  |  |
| 1475 | CSTA | cystatin A (stefin A) | 3q21 | - [MF] GO:0004869: cysteine protease inhibitor activity - [CC] GO:0005622: intracellular \* | - pfam00031: Cystatin domain |  |  |
| 6678 | SPARC | secreted protein, acidic, cysteine-rich (osteonectin) | 5q31.3-q32 | - [BP] GO:0001503: ossification - [MF] GO:0005509: calcium ion binding \* - [MF] GO:0005518: collagen binding \* - [CC] GO:0005604: basement membrane | - cd00104: Kazal type serine protease inhibitors and follistatin-like domains - cd00252: SPARC\_EC - smart00274: Follistatin-N-terminal domain-like |  |  |
| 2171 | FABP5 | fatty acid binding protein 5 (psoriasis-associated) | 8q21.13 | - [MF] GO:0005215: transporter activity \* - [MF] GO:0005504: fatty acid binding - [CC] GO:0005737: cytoplasm \* - [BP] GO:0006629: lipid metabolism - [BP] GO:0006810: transport \* - [BP] GO:0008544: epidermis development \* | - KOG4015: Fatty acid-binding protein FABP [Lipid transport and metabolism] |  |  |
| 8323 | FZD6 | frizzled homolog 6 (Drosophila) | 8q22.3-q23.1 |  | - pfam01534: Frizzled/Smoothened family membrane region - smart00063: Frizzled | - Wnt signaling pathway |  |
| 10397 | NDRG1 | N-myc downstream regulated gene 1 | 8q24.3 | - [MF] GO:0003824: catalytic activity \* - [CC] GO:0005634: nucleus \* - [BP] GO:0010038: response to metal ion - [BP] GO:0030154: cell differentiation \* | - KOG2931: Differentiation-related gene 1 protein (NDR1 protein), related proteins [Function unknown] |  | - 601455 |
| 6836 | SURF4 | surfeit 4 | 9q34.2 | - [BP] GO:0000004: biological\_process unknown - [MF] GO:0005554: molecular\_function unknown - [CC] GO:0005789: endoplasmic reticulum membrane - [CC] GO:0016021: integral to membrane \* | - pfam02077: SURF4 family |  |  |
| 1646 | AKR1C2 | aldo-keto reductase family 1, member C2 (dihydrodiol dehydrogenase 2; bile acid binding protein; 3-alpha hydroxysteroid dehydrogenase, type III) | 10p15-p14 | - [MF] GO:0005488: binding \* - [MF] GO:0005489: electron transporter activity \* - [BP] GO:0006629: lipid metabolism - [BP] GO:0006810: transport \* - [BP] GO:0007586: digestion - [MF] GO:0015125: bile acid transporter activity - [BP] GO:0015722: canalicular bile acid transport - [MF] GO:0016491: oxidoreductase activity \* - [MF] GO:0047115: trans-1,2-dihydrobenzene-1,2-diol dehydrogenase activity | - KOG1577: Aldo/keto reductase family proteins [General function prediction only] |  |  |
| 3185 | HNRPF | heterogeneous nuclear ribonucleoprotein F | 10q11.21-q11.22 | - [MF] GO:0003723: RNA binding \* - [BP] GO:0006396: RNA processing - [CC] GO:0030530: heterogeneous nuclear ribonucleoprotein complex | - KOG4211: Splicing factor hnRNP-F and related RNA-binding proteins [RNA processing and modification] - smart00360: RNA recognition motif |  |  |
| 9446 | GSTO1 | glutathione S-transferase omega 1 | 10q25.1 | - [MF] GO:0004364: glutathione transferase activity - [CC] GO:0005737: cytoplasm \* - [BP] GO:0008152: metabolism \* - [MF] GO:0016656: monodehydroascorbate reductase (NADH) activity - [MF] GO:0016740: transferase activity \* | - KOG0406: Glutathione S-transferase [Posttranslational modification, protein turnover, chaperones] | - Glutathione metabolism |  |
| 8495 | PPFIBP2 | PTPRF interacting protein, binding protein 2 (liprin beta 2) | 11p15.4 |  | - cd00166: Sterile alpha motif. - COG0419: ATPase involved in DNA repair [DNA replication, recombination, and repair] - COG1196: Chromosome segregation ATPases [Cell division and chromosome partitioning] - COG2433: Uncharacterized conserved protein [Function unknown] - pfam00536: SAM domain (Sterile alpha motif). It has been suggested that SAM is an evolutionarily conserved protein binding domain that is involved in the regulation of numerous developmental processes in diverse eukaryotes. The SAM domain can potentially function as - pfam02920: DNA binding domain of tn916 integrase - smart00454: Sterile alpha motif |  |  |
| 27122 | DKK3 | dickkopf homolog 3 (Xenopus laevis) | 11p15.2 | - [CC] GO:0005576: extracellular \* - [CC] GO:0005615: extracellular space \* - [BP] GO:0007275: development \* - [BP] GO:0009653: morphogenesis \* - [BP] GO:0016055: Wnt receptor signaling pathway - [BP] GO:0030178: negative regulation of Wnt receptor signaling pathway | - pfam04706: Dickkopf N-terminal cysteine-rich region - pfam06607: Prokineticin |  |  |
| 392 | ARHGAP1 | Rho GTPase activating protein 1 | 11p12-q12 | - [MF] GO:0005070: SH3/SH2 adaptor protein activity - [MF] GO:0005100: Rho GTPase activator activity - [MF] GO:0005100: Rho GTPase activator activity - [MF] GO:0005525: GTP binding \* - [BP] GO:0007010: cytoskeleton organization and biogenesis - [BP] GO:0007165: signal transduction \* - [BP] GO:0007266: Rho protein signal transduction - [CC] GO:0008372: cellular\_component unknown | - cd00159: GTPase-activator protein for Rho-like GTPases - cd00170: Sec14p-like lipid-binding domain |  |  |
| 2950 | GSTP1 | glutathione S-transferase pi | 11q13 | - [MF] GO:0004364: glutathione transferase activity - [BP] GO:0007417: central nervous system development - [BP] GO:0008152: metabolism \* - [MF] GO:0016740: transferase activity \* | - KOG1695: Glutathione S-transferase [Posttranslational modification, protein turnover, chaperones] | - Glutathione metabolism |  |
| 871 | SERPINH1 | serine (or cysteine) proteinase inhibitor, clade H (heat shock protein 47), member 1, (collagen binding protein 1) | 11q13.5 | - [MF] GO:0003773: heat shock protein activity - [MF] GO:0004867: serine-type endopeptidase inhibitor activity - [MF] GO:0004867: serine-type endopeptidase inhibitor activity - [MF] GO:0005518: collagen binding \* - [CC] GO:0005783: endoplasmic reticulum \* - [CC] GO:0005783: endoplasmic reticulum \* - [BP] GO:0006950: response to stress | - pfam00079: Serpin (serine protease inhibitor) |  |  |
| 2597 | GAPD | glyceraldehyde-3-phosphate dehydrogenase | 12p13 | - [MF] GO:0004365: glyceraldehyde-3-phosphate dehydrogenase (phosphorylating) activity - [CC] GO:0005737: cytoplasm \* - [BP] GO:0006006: glucose metabolism - [BP] GO:0006096: glycolysis \* - [MF] GO:0016491: oxidoreductase activity \* | - KOG0657: Glyceraldehyde 3-phosphate dehydrogenase [Carbohydrate transport and metabolism] | - Glycolysis / Gluconeogenesis - D-Arginine and D-ornithine metabolism - Neurodegenerative Disorders - Alzheimer's disease - Huntington's disease - Dentatorubropallidoluysian atrophy (DRPLA) |  |
| 5744 | PTHLH | parathyroid hormone-like hormone | 12p12.1-p11.2 | - [MF] GO:0005179: hormone activity - [CC] GO:0005615: extracellular space \* - [CC] GO:0005737: cytoplasm \* - [BP] GO:0007267: cell-cell signaling - [BP] GO:0007565: pregnancy - [BP] GO:0007595: lactation - [BP] GO:0008284: positive regulation of cell proliferation - [BP] GO:0008285: negative regulation of cell proliferation - [BP] GO:0008544: epidermis development \* - [BP] GO:0046058: cAMP metabolism | - pfam01279: Parathyroid hormone family |  |  |
| 3848 | KRT1 | keratin 1 (epidermolytic hyperkeratosis) | 12q12-q13 | - [MF] GO:0005200: structural constituent of cytoskeleton - [CC] GO:0005856: cytoskeleton - [CC] GO:0005882: intermediate filament - [BP] GO:0008544: epidermis development \* | - pfam00038: Intermediate filament protein |  | - 113800 - 146590 - 148700 - 600962 - 607602 - 607654 |
| 6472 | SHMT2 | serine hydroxymethyltransferase 2 (mitochondrial) | 12q12-q14 | - [MF] GO:0004372: glycine hydroxymethyltransferase activity - [CC] GO:0005739: mitochondrion \* - [BP] GO:0006544: glycine metabolism - [BP] GO:0006563: L-serine metabolism - [BP] GO:0006730: one-carbon compound metabolism - [MF] GO:0016740: transferase activity \* | - pfam00464: Serine hydroxymethyltransferase | - Glycine, serine and threonine metabolism - Lysine degradation - Cyanoamino acid metabolism - One carbon pool by folate - Methane metabolism |  |
| 1466 | CSRP2 | cysteine and glycine-rich protein 2 | 12q21.1 | - [MF] GO:0005554: molecular\_function unknown - [CC] GO:0005634: nucleus \* - [BP] GO:0007517: muscle development - [BP] GO:0008283: cell proliferation \* - [BP] GO:0016049: cell growth - [BP] GO:0030154: cell differentiation \* | - smart00132: Zinc-binding domain present in Lin-11, Isl-1, Mec-3 |  |  |
| 8892 | EIF2B2 | eukaryotic translation initiation factor 2B, subunit 2 beta, 39kDa | 14q24.3 | - [MF] GO:0003743: translation initiation factor activity - [MF] GO:0005085: guanyl-nucleotide exchange factor activity - [MF] GO:0005525: GTP binding \* - [CC] GO:0005851: eukaryotic translation initiation factor 2B complex - [BP] GO:0006412: protein biosynthesis - [BP] GO:0006413: translational initiation | - KOG1465: Translation initiation factor 2B, beta subunit (eIF-2Bbeta/GCD7) [Translation, ribosomal structure and biogenesis] |  | - 603896 |
| 5315 | PKM2 | pyruvate kinase, muscle | 15q22 | - [MF] GO:0000287: magnesium ion binding \* - [MF] GO:0004743: pyruvate kinase activity - [CC] GO:0005829: cytosol - [BP] GO:0006096: glycolysis \* - [MF] GO:0016740: transferase activity \* | - KOG2323: Pyruvate kinase [Carbohydrate transport and metabolism] | - Glycolysis / Gluconeogenesis - Purine metabolism - Pyruvate metabolism - Carbon fixation |  |
| 4490 | MT1B | metallothionein 1B (functional) | 16q13 | - [BP] GO:0000004: biological\_process unknown - [MF] GO:0005507: copper ion binding \* - [CC] GO:0005737: cytoplasm \* - [MF] GO:0008270: zinc ion binding \* - [MF] GO:0046870: cadmium ion binding - [MF] GO:0046872: metal ion binding \* |  |  |  |
| 4494 | MT1F | metallothionein 1F (functional) | 16q13 | - [BP] GO:0000004: biological\_process unknown - [MF] GO:0005507: copper ion binding \* - [CC] GO:0005737: cytoplasm \* - [MF] GO:0008270: zinc ion binding \* - [MF] GO:0046870: cadmium ion binding - [MF] GO:0046872: metal ion binding \* |  |  |  |
| 4495 | MT1G | metallothionein 1G | 16q13 | - [MF] GO:0046872: metal ion binding \* |  |  |  |
| 4499 | MT1K | metallothionein 1K | 16q13 | - [MF] GO:0046872: metal ion binding \* |  |  |  |
| 4501 | MT1X | metallothionein 1X | 16q13 | - [BP] GO:0010038: response to metal ion - [MF] GO:0046872: metal ion binding \* |  |  |  |
| 4504 | MT3 | metallothionein 3 (growth inhibitory factor (neurotrophic)) | 16q13 | - [BP] GO:0001666: response to hypoxia - [MF] GO:0005489: electron transporter activity \* - [MF] GO:0005506: iron ion binding - [MF] GO:0005507: copper ion binding \* - [BP] GO:0006118: electron transport - [BP] GO:0006875: metal ion homeostasis - [CC] GO:0008021: synaptic vesicle - [MF] GO:0008270: zinc ion binding \* - [BP] GO:0008283: cell proliferation \* - [MF] GO:0016209: antioxidant activity - [BP] GO:0019430: removal of superoxide radicals - [BP] GO:0030308: negative regulation of cell growth - [MF] GO:0046872: metal ion binding \* - [BP] GO:0050774: negative regulation of dendrite morphogenesis |  |  |  |
| 1277 | COL1A1 | collagen, type I, alpha 1 | 17q21.3-q22.1 | - [BP] GO:0001501: skeletal development \* - [MF] GO:0005201: extracellular matrix structural constituent - [CC] GO:0005581: collagen - [CC] GO:0005584: collagen type I - [CC] GO:0005737: cytoplasm \* - [BP] GO:0006817: phosphate transport - [BP] GO:0007605: perception of sound - [MF] GO:0008147: structural constituent of bone - [BP] GO:0008544: epidermis development \* | - KOG3546: Collagens (type XV) [Extracellular structures] - pfam00093: von Willebrand factor type C domain - pfam01410: Fibrillar collagen C-terminal domain |  | - 120150 - 130000 - 130060 - 166200 - 166210 - 166220 - 166710 - 259420 |
| 284119 | PTRF | polymerase I and transcript release factor | 17q21.31 |  |  |  |  |
| 3613 | IMPA2 | inositol(myo)-1(or 4)-monophosphatase 2 | 18p11.2 | - [MF] GO:0000287: magnesium ion binding \* - [BP] GO:0006796: phosphate metabolism - [BP] GO:0007165: signal transduction \* - [MF] GO:0008934: inositol-1(or 4)-monophosphatase activity - [MF] GO:0016787: hydrolase activity \* | - pfam00459: Inositol monophosphatase family | - Streptomycin biosynthesis - Inositol phosphate metabolism - Phosphatidylinositol signaling system |  |
| 5268 | SERPINB5 | serine (or cysteine) proteinase inhibitor, clade B (ovalbumin), member 5 | 18q21.3 | - [MF] GO:0004867: serine-type endopeptidase inhibitor activity - [MF] GO:0004867: serine-type endopeptidase inhibitor activity - [BP] GO:0006928: cell motility | - pfam00079: Serpin (serine protease inhibitor) |  |  |
| 7386 | UQCRFS1 | ubiquinol-cytochrome c reductase, Rieske iron-sulfur polypeptide 1 | 19q12-q13.1 | - [CC] GO:0005739: mitochondrion \* - [BP] GO:0006118: electron transport - [MF] GO:0008121: ubiquinol-cytochrome-c reductase activity - [CC] GO:0015008: ubiquinol-cytochrome-c reductase complex (sensu Eukarya) - [CC] GO:0016021: integral to membrane \* - [MF] GO:0016491: oxidoreductase activity \* - [CC] GO:0019866: inner membrane | - KOG1671: Ubiquinol cytochrome c reductase, subunit RIP1 [Energy production and conversion] | - Oxidative phosphorylation - Electron Transport System, Complex III |  |
| 2091 | FBL | fibrillarin | 19q13.1 | - [MF] GO:0003723: RNA binding \* - [CC] GO:0005634: nucleus \* - [BP] GO:0006364: rRNA processing - [CC] GO:0030529: ribonucleoprotein complex | - KOG1596: Fibrillarin and related nucleolar RNA-binding proteins [RNA processing and modification] |  |  |
| 2821 | GPI | glucose phosphate isomerase | 19q13.1 | - [MF] GO:0004347: glucose-6-phosphate isomerase activity - [MF] GO:0005125: cytokine activity - [BP] GO:0005975: carbohydrate metabolism - [BP] GO:0006094: gluconeogenesis - [BP] GO:0006096: glycolysis \* - [BP] GO:0006959: humoral immune response - [BP] GO:0007399: neurogenesis \* - [BP] GO:0007599: hemostasis - [MF] GO:0008083: growth factor activity - [MF] GO:0016853: isomerase activity | - KOG2446: Glucose-6-phosphate isomerase [Carbohydrate transport and metabolism] | - Glycolysis / Gluconeogenesis - Pentose phosphate pathway - Starch and sucrose metabolism | - 172400 |
| 5621 | PRNP | prion protein (p27-30) (Creutzfeld-Jakob disease, Gerstmann-Strausler-Scheinker syndrome, fatal familial insomnia) | 20pter-p12 | - [BP] GO:0008152: metabolism \* | - smart00157: Major prion protein | - Neurodegenerative Disorders - Prion disease | - 123400 - 137440 - 600072 - 603218 - 606688 |
| 10904 | BLCAP | bladder cancer associated protein | 20q11.2-q12 | - [BP] GO:0000004: biological\_process unknown - [MF] GO:0005554: molecular\_function unknown - [CC] GO:0016021: integral to membrane \* | - KOG4489: Uncharacterized conserved protein BC10 (implicated in bladder cancer in humans) [Function unknown] |  |  |
| 873 | CBR1 | carbonyl reductase 1 | 21q22.13 |  | - KOG1208: Dehydrogenases with different specificities (related to short-chain alcohol dehydrogenases) [Secondary metabolites biosynthesis, transport and catabolism] | - Prostaglandin and leukotriene metabolism |  |
| 7078 | TIMP3 | tissue inhibitor of metalloproteinase 3 (Sorsby fundus dystrophy, pseudoinflammatory) | 22q12.1-q13.2 | - [CC] GO:0005578: extracellular matrix \* - [CC] GO:0005578: extracellular matrix \* - [BP] GO:0007601: visual perception - [MF] GO:0008191: metalloendopeptidase inhibitor activity - [BP] GO:0008624: induction of apoptosis by extracellular signals | - smart00206: Tissue inhibitor of metalloproteinase family |  | - 136900 |
| 23481 | PES1 | pescadillo homolog 1, containing BRCT domain (zebrafish) | 22q12.1 | - [CC] GO:0005634: nucleus \* - [BP] GO:0009653: morphogenesis \* | - COG5163: Protein required for biogenesis of the 60S ribosomal subunit [Translation, ribosomal structure and biogenesis] - KOG2481: Protein required for normal rRNA processing [RNA processing and modification] |  |  |

# Class 1 (17 LocusIDs)

| LocusLink | Symbol | Name | Cytoband Location | GO Terms | Domain Terms | KEGG Pathway | OMIM |
| --- | --- | --- | --- | --- | --- | --- | --- |
| 6401 | SELE | selectin E (endothelial adhesion molecule 1) | 1q22-q25 | - [MF] GO:0005515: protein binding \* - [MF] GO:0005529: sugar binding - [CC] GO:0005886: plasma membrane \* - [BP] GO:0006954: inflammatory response - [BP] GO:0007155: cell adhesion \* - [BP] GO:0007157: heterophilic cell adhesion - [CC] GO:0016021: integral to membrane \* | - cd00033: Domain abundant in complement control proteins - cd00054: Calcium-binding EGF-like domain, present in a large number of membrane-bound and extracellular (mostly animal) proteins - KOG4289: Cadherin EGF LAG seven-pass G-type receptor [Signal transduction mechanisms] - pfam00059: Lectin C-type domain |  | - 131210 |
| 3655 | ITGA6 | integrin, alpha 6 | 2q31.1 | - [MF] GO:0004872: receptor activity \* - [MF] GO:0005515: protein binding \* - [BP] GO:0007044: cell-substrate junction assembly - [BP] GO:0007160: cell-matrix adhesion \* - [BP] GO:0007229: integrin-mediated signaling pathway - [CC] GO:0008305: integrin complex - [CC] GO:0016021: integral to membrane \* | - KOG3637: Vitronectin receptor, alpha subunit [Extracellular structures] - smart00191: Integrin alpha (beta-propellor repeats) |  | - 226730 |
| 2817 | GPC1 | glypican 1 | 2q35-q37 | - [MF] GO:0004888: transmembrane receptor activity - [CC] GO:0005578: extracellular matrix \* - [CC] GO:0005615: extracellular space \* - [CC] GO:0005887: integral to plasma membrane \* - [BP] GO:0007275: development \* - [CC] GO:0016020: membrane \* | - KOG3821: Heparin sulfate cell surface proteoglycan [Signal transduction mechanisms] |  |  |
| 483 | ATP1B3 | ATPase, Na+/K+ transporting, beta 3 polypeptide | 3q23 | - [MF] GO:0005391: sodium/potassium-exchanging ATPase activity - [CC] GO:0005890: sodium/potassium-exchanging ATPase complex - [BP] GO:0006810: transport \* - [BP] GO:0006813: potassium ion transport - [BP] GO:0006814: sodium ion transport - [CC] GO:0016021: integral to membrane \* | - pfam00287: Sodium / potassium ATPase beta chain | - Oxidative phosphorylation |  |
| 10296 | MAEA | macrophage erythroblast attacher | 4p16.3 | - [CC] GO:0005624: membrane fraction \* - [CC] GO:0005887: integral to plasma membrane \* - [BP] GO:0006915: apoptosis \* - [BP] GO:0007155: cell adhesion \* - [BP] GO:0007275: development \* | - KOG0396: Uncharacterized conserved protein [Function unknown] |  |  |
| 374 | AREG | amphiregulin (schwannoma-derived growth factor) | 4q13-q21 | - [MF] GO:0005125: cytokine activity - [CC] GO:0005615: extracellular space \* - [BP] GO:0007267: cell-cell signaling - [MF] GO:0008083: growth factor activity \* - [BP] GO:0008283: cell proliferation \* - [CC] GO:0016021: integral to membrane \* |  |  |  |
| 26025 | PCDHGA12 | protocadherin gamma subfamily A, 12 | 5q31 | - [MF] GO:0005509: calcium ion binding \* - [MF] GO:0005515: protein binding \* - [BP] GO:0007155: cell adhesion \* - [BP] GO:0007156: homophilic cell adhesion \* - [CC] GO:0016021: integral to membrane \* | - cd00031: Cadherin repeat domain - KOG4289: Cadherin EGF LAG seven-pass G-type receptor [Signal transduction mechanisms] |  |  |
| 2196 | FAT2 | FAT tumor suppressor homolog 2 (Drosophila) | 5q32-q33 | - [MF] GO:0005198: structural molecule activity \* - [MF] GO:0005509: calcium ion binding \* - [MF] GO:0005515: protein binding \* - [BP] GO:0007155: cell adhesion \* - [BP] GO:0007156: homophilic cell adhesion \* - [CC] GO:0016021: integral to membrane \* | - cd00031: Cadherin repeat domain - cd00054: Calcium-binding EGF-like domain, present in a large number of membrane-bound and extracellular (mostly animal) proteins - cd00110: Laminin G domain - smart00112: Cadherin repeats |  |  |
| 857 | CAV1 | caveolin 1, caveolae protein, 22kDa | 7q31.1 | - [MF] GO:0005198: structural molecule activity \* - [CC] GO:0005887: integral to plasma membrane \* - [CC] GO:0016599: caveolar membrane | - pfam01146: Caveolin | - Integrin-mediated cell adhesion |  |
| 960 | CD44 | CD44 antigen (homing function and Indian blood group system) | 11p13 | - [MF] GO:0004872: receptor activity \* - [MF] GO:0005518: collagen binding - [MF] GO:0005540: hyaluronic acid binding - [MF] GO:0005540: hyaluronic acid binding - [CC] GO:0005887: integral to plasma membrane \* - [BP] GO:0007155: cell adhesion \* - [BP] GO:0007160: cell-matrix adhesion \* - [CC] GO:0016020: membrane \* - [BP] GO:0016337: cell-cell adhesion \* | - smart00445: Link (Hyaluronan-binding) |  |  |
| 1012 | CDH13 | cadherin 13, H-cadherin (heart) | 16q24.2-q24.3 | - [MF] GO:0005509: calcium ion binding \* - [MF] GO:0005515: protein binding \* - [BP] GO:0007155: cell adhesion \* - [BP] GO:0007156: homophilic cell adhesion \* - [CC] GO:0016020: membrane \* | - cd00031: Cadherin repeat domain - KOG4289: Cadherin EGF LAG seven-pass G-type receptor [Signal transduction mechanisms] |  |  |
| 57555 | NLGN2 | neuroligin 2 | 17p13.2 | - [MF] GO:0003824: catalytic activity \* - [BP] GO:0007416: synaptogenesis - [CC] GO:0016021: integral to membrane \* - [BP] GO:0016337: cell-cell adhesion \* - [MF] GO:0042043: neurexin binding - [CC] GO:0045211: postsynaptic membrane - [BP] GO:0045217: intercellular junction maintenance | - pfam00135: Carboxylesterase |  |  |
| 3675 | ITGA3 | integrin, alpha 3 (antigen CD49C, alpha 3 subunit of VLA-3 receptor) | 17q21.33 | - [MF] GO:0004872: receptor activity \* - [MF] GO:0005515: protein binding \* - [BP] GO:0007160: cell-matrix adhesion \* - [BP] GO:0007229: integrin-mediated signaling pathway - [CC] GO:0008305: integrin complex - [CC] GO:0016021: integral to membrane \* | - smart00191: Integrin alpha (beta-propellor repeats) |  |  |
| 3963 | LGALS7 | lectin, galactoside-binding, soluble, 7 (galectin 7) | 19q13.2 | - [MF] GO:0005529: sugar binding - [CC] GO:0005615: extracellular space \* - [CC] GO:0005634: nucleus - [BP] GO:0006915: apoptosis \* - [BP] GO:0007157: heterophilic cell adhesion - [BP] GO:0008151: cell growth and/or maintenance \* | - smart00276: Galectin |  |  |
| 6510 | SLC1A5 | solute carrier family 1 (neutral amino acid transporter), member 5 | 19q13.3 | - [MF] GO:0004872: receptor activity \* - [CC] GO:0005624: membrane fraction \* - [CC] GO:0005887: integral to plasma membrane \* - [BP] GO:0006810: transport \* - [BP] GO:0006835: dicarboxylic acid transport - [MF] GO:0015175: neutral amino acid transporter activity - [MF] GO:0015293: symporter activity - [BP] GO:0015804: neutral amino acid transport - [MF] GO:0017153: sodium:dicarboxylate symporter activity | - KOG3787: Glutamate/aspartate and neutral amino acid transporters [Amino acid transport and metabolism] |  |  |
| 182 | JAG1 | jagged 1 (Alagille syndrome) | 20p12.1-p11.23 | - [BP] GO:0001525: angiogenesis - [BP] GO:0001709: cell fate determination - [MF] GO:0005112: Notch binding - [MF] GO:0005198: structural molecule activity \* - [MF] GO:0005509: calcium ion binding \* - [MF] GO:0005509: calcium ion binding \* - [CC] GO:0005576: extracellular \* - [CC] GO:0005887: integral to plasma membrane \* - [BP] GO:0007154: cell communication \* - [BP] GO:0007219: Notch signaling pathway - [BP] GO:0007275: development \* - [BP] GO:0007399: neurogenesis \* - [MF] GO:0008083: growth factor activity \* - [BP] GO:0030097: hemopoiesis - [BP] GO:0030216: keratinocyte differentiation - [BP] GO:0030334: regulation of cell migration - [BP] GO:0042127: regulation of cell proliferation - [BP] GO:0045445: myoblast differentiation - [BP] GO:0045446: endothelial cell differentiation | - cd00054: Calcium-binding EGF-like domain, present in a large number of membrane-bound and extracellular (mostly animal) proteins - KOG1217: Fibrillins and related proteins containing Ca2+-binding EGF-like domains [Signal transduction mechanisms] - KOG1219: Uncharacterized conserved protein, contains laminin, cadherin and EGF domains [Signal transduction mechanisms] - pfam01414: Delta serrate ligand - smart00215: von Willebrand factor (vWF) type C domain |  | - 118450 - 187500 - 601920 |
| 5355 | PLP2 | proteolipid protein 2 (colonic epithelium-enriched) | Xp11.23 | - [CC] GO:0005624: membrane fraction \* - [CC] GO:0005789: endoplasmic reticulum membrane - [BP] GO:0006811: ion transport - [MF] GO:0015075: ion transporter activity - [CC] GO:0016021: integral to membrane \* | - KOG4788: Members of chemokine-like factor super family and related proteins [Defense mechanisms] |  |  |

# Class 2 (15 LocusIDs)

| LocusLink | Symbol | Name | Cytoband Location | GO Terms | Domain Terms | KEGG Pathway | OMIM |
| --- | --- | --- | --- | --- | --- | --- | --- |
| 2810 | SFN | stratifin | 1p35.3 | - [BP] GO:0000074: regulation of cell cycle \* - [CC] GO:0005615: extracellular space \* - [CC] GO:0005737: cytoplasm \* - [BP] GO:0006469: negative regulation of protein kinase activity - [BP] GO:0007165: signal transduction \* - [BP] GO:0008283: cell proliferation \* - [MF] GO:0008426: protein kinase C inhibitor activity - [MF] GO:0019904: protein domain specific binding | - smart00101: 14-3-3 homologues |  |  |
| 4638 | MYLK | myosin, light polypeptide kinase | 3q21 | - [MF] GO:0004674: protein serine/threonine kinase activity - [MF] GO:0004687: myosin-light-chain kinase activity - [MF] GO:0004871: signal transducer activity \* - [MF] GO:0005516: calmodulin binding - [MF] GO:0005524: ATP binding \* - [BP] GO:0006468: protein amino acid phosphorylation \* - [BP] GO:0006468: protein amino acid phosphorylation \* - [MF] GO:0016301: kinase activity - [MF] GO:0016740: transferase activity \* | - cd00063: Fibronectin type 3 domain - cd00180: Serine/Threonine protein kinases, catalytic domain - cd00931: Immunoglobulin domain cell adhesion molecule (cam) subfamily |  |  |
| 1452 | CSNK1A1 | casein kinase 1, alpha 1 | 5q32 | - [MF] GO:0004674: protein serine/threonine kinase activity - [MF] GO:0004681: casein kinase I activity - [MF] GO:0005524: ATP binding \* - [BP] GO:0006468: protein amino acid phosphorylation \* - [BP] GO:0016055: Wnt receptor signaling pathway - [MF] GO:0016740: transferase activity \* | - cd00180: Serine/Threonine protein kinases, catalytic domain | - Starch and sucrose metabolism - Inositol phosphate metabolism - Sphingoglycolipid metabolism - Benzoate degradation via CoA ligation - Nicotinate and nicotinamide metabolism - Porphyrin and chlorophyll metabolism - Wnt signaling pathway - Circadian rhythm |  |
| 5644 | PRSS1 | protease, serine, 1 (trypsin 1) | 7q32-qter | - [MF] GO:0004263: chymotrypsin activity - [MF] GO:0004295: trypsin activity \* - [MF] GO:0005509: calcium ion binding \* - [CC] GO:0005576: extracellular \* - [BP] GO:0006508: proteolysis and peptidolysis \* - [BP] GO:0007586: digestion - [MF] GO:0016787: hydrolase activity \* | - cd00190: Trypsin-like serine protease |  | - 167800 - 276000 |
| 5327 | PLAT | plasminogen activator, tissue | 8p12 | - [MF] GO:0004263: chymotrypsin activity - [MF] GO:0004295: trypsin activity \* - [CC] GO:0005576: extracellular \* - [BP] GO:0006464: protein modification \* - [BP] GO:0006508: proteolysis and peptidolysis \* - [BP] GO:0006508: proteolysis and peptidolysis \* - [BP] GO:0007596: blood coagulation - [MF] GO:0008243: plasminogen activator activity - [MF] GO:0016787: hydrolase activity \* | - cd00190: Trypsin-like serine protease - KOG1219: Uncharacterized conserved protein, contains laminin, cadherin and EGF domains [Signal transduction mechanisms] - pfam00051: Kringle domain - smart00058: Fibronectin type 1 domain | - Coagulation cascade | - 173370 |
| 1514 | CTSL | cathepsin L | 9q21-q22 | - [MF] GO:0004217: cathepsin L activity - [CC] GO:0005576: extracellular \* - [CC] GO:0005764: lysosome - [BP] GO:0006508: proteolysis and peptidolysis \* - [MF] GO:0016787: hydrolase activity \* | - KOG1543: Cysteine proteinase Cathepsin L [Posttranslational modification, protein turnover, chaperones] |  |  |
| 5654 | PRSS11 | protease, serine, 11 (IGF binding) | 10q26.3 | - [BP] GO:0001558: regulation of cell growth - [MF] GO:0004295: trypsin activity \* - [MF] GO:0005520: insulin-like growth factor binding - [CC] GO:0005615: extracellular space \* - [BP] GO:0006508: proteolysis and peptidolysis \* - [BP] GO:0008151: cell growth and/or maintenance \* - [MF] GO:0016787: hydrolase activity \* | - COG0265: Trypsin-like serine proteases, typically periplasmic, contain C-terminal PDZ domain [Posttranslational modification, protein turnover, chaperones] - KOG1320: Serine protease [Posttranslational modification, protein turnover, chaperones] - smart00121: Insulin growth factor-binding protein homologues |  |  |
| 1075 | CTSC | cathepsin C | 11q14.1-q14.3 | - [MF] GO:0004197: cysteine-type endopeptidase activity - [MF] GO:0004214: dipeptidyl-peptidase I activity - [CC] GO:0005764: lysosome - [BP] GO:0006508: proteolysis and peptidolysis \* - [BP] GO:0006955: immune response - [MF] GO:0016787: hydrolase activity \* | - pfam00112: Papain family cysteine protease |  | - 170650 - 245000 - 245010 |
| 4314 | MMP3 | matrix metalloproteinase 3 (stromelysin 1, progelatinase) | 11q22.3 | - [MF] GO:0004248: stromelysin 1 activity - [MF] GO:0005509: calcium ion binding \* - [CC] GO:0005578: extracellular matrix \* - [CC] GO:0005615: extracellular space \* - [MF] GO:0008270: zinc ion binding - [MF] GO:0016787: hydrolase activity \* - [BP] GO:0030574: collagen catabolism | - cd00094: Hemopexin-like repeats - KOG1565: Gelatinase A and related matrix metalloproteases [Posttranslational modification, protein turnover, chaperones, Extracellular structures] - pfam00413: Matrixin - pfam03933: Matrix metalloprotease, N-terminal domain |  | - 185250 |
| 10576 | CCT2 | chaperonin containing TCP1, subunit 2 (beta) | 12q14.3 | - [BP] GO:0000074: regulation of cell cycle \* - [MF] GO:0003754: chaperone activity - [MF] GO:0005524: ATP binding \* - [CC] GO:0005737: cytoplasm \* - [BP] GO:0006457: protein folding | - COG0459: Chaperonin GroEL (HSP60 family) [Posttranslational modification, protein turnover, chaperones] - KOG0363: Chaperonin complex component, TCP-1 beta subunit (CCT2) [Posttranslational modification, protein turnover, chaperones] |  |  |
| 1848 | DUSP6 | dual specificity phosphatase 6 | 12q22-q23 | - [BP] GO:0000074: regulation of cell cycle \* - [BP] GO:0000188: inactivation of MAPK - [MF] GO:0004722: protein serine/threonine phosphatase activity - [MF] GO:0004725: protein tyrosine phosphatase activity - [CC] GO:0005625: soluble fraction - [CC] GO:0005737: cytoplasm \* - [BP] GO:0006470: protein amino acid dephosphorylation - [MF] GO:0016787: hydrolase activity \* - [MF] GO:0017017: MAP kinase phosphatase activity | - cd00127: Dual specificity phosphatases (DSP) - cd00158: Rhodanese Homology Domain - KOG1717: Dual specificity phosphatase [Defense mechanisms] - smart00450: Rhodanese Homology Domain | - MAPK signaling pathway - Phosphatidylinositol signaling system |  |
| 5901 | RAN | RAN, member RAS oncogene family | 12q24.3 | - [BP] GO:0000074: regulation of cell cycle \* - [CC] GO:0000785: chromatin - [MF] GO:0003924: GTPase activity - [MF] GO:0005525: GTP binding - [CC] GO:0005634: nucleus - [CC] GO:0005643: nuclear pore - [BP] GO:0006259: DNA metabolism - [BP] GO:0006405: RNA-nucleus export - [BP] GO:0006611: protein-nucleus export - [BP] GO:0006886: intracellular protein transport - [BP] GO:0007052: mitotic spindle assembly - [BP] GO:0007165: signal transduction \* - [BP] GO:0007264: small GTPase mediated signal transduction | - smart00176: Ran (Ras-related nuclear proteins) /TC4 subfamily of small GTPases |  |  |
| 3480 | IGF1R | insulin-like growth factor 1 receptor | 15q26.3 | - [BP] GO:0000074: regulation of cell cycle \* - [MF] GO:0004872: receptor activity - [MF] GO:0005006: epidermal growth factor receptor activity - [MF] GO:0005010: insulin-like growth factor receptor activity - [MF] GO:0005515: protein binding \* - [MF] GO:0005524: ATP binding \* - [BP] GO:0006468: protein amino acid phosphorylation \* - [BP] GO:0006916: anti-apoptosis - [BP] GO:0007165: signal transduction \* - [BP] GO:0008284: positive regulation of cell proliferation \* - [BP] GO:0008286: insulin receptor signaling pathway - [CC] GO:0016021: integral to membrane \* - [MF] GO:0016740: transferase activity \* | - cd00063: Fibronectin type 3 domain - cd00192: Tyrosine kinase, catalytic domain - KOG4258: Insulin/growth factor receptor (contains protein kinase domain) [Signal transduction mechanisms] - pfam00757: Furin-like cysteine rich region - pfam01030: Receptor L domain |  | - 147370 |
| 1973 | EIF4A1 | eukaryotic translation initiation factor 4A, isoform 1 | 17p13 | - [MF] GO:0003676: nucleic acid binding - [MF] GO:0003677: DNA binding - [MF] GO:0003723: RNA binding - [MF] GO:0003743: translation initiation factor activity - [MF] GO:0005524: ATP binding \* - [BP] GO:0006412: protein biosynthesis - [MF] GO:0008026: ATP-dependent helicase activity - [MF] GO:0016787: hydrolase activity \* | - KOG0327: Translation initiation factor 4F, helicase subunit (eIF-4A) and related helicases [Translation, ribosomal structure and biogenesis] |  |  |
| 7076 | TIMP1 | tissue inhibitor of metalloproteinase 1 (erythroid potentiating activity, collagenase inhibitor) | Xp11.3-p11.23 | - [CC] GO:0005578: extracellular matrix \* - [BP] GO:0006508: proteolysis and peptidolysis \* - [BP] GO:0007275: development \* - [MF] GO:0008191: metalloendopeptidase inhibitor activity - [BP] GO:0008284: positive regulation of cell proliferation \* | - pfam00965: Tissue inhibitor of metalloproteinase |  |  |

# Class 3 (9 LocusIDs)

| LocusLink | Symbol | Name | Cytoband Location | GO Terms | Domain Terms | KEGG Pathway | OMIM |
| --- | --- | --- | --- | --- | --- | --- | --- |
| 2034 | EPAS1 | endothelial PAS domain protein 1 | 2p21-p16 | - [BP] GO:0001525: angiogenesis - [MF] GO:0003705: RNA polymerase II transcription factor activity, enhancer binding - [MF] GO:0003713: transcription coactivator activity - [MF] GO:0004871: signal transducer activity - [CC] GO:0005634: nucleus \* - [BP] GO:0006355: regulation of transcription, DNA-dependent \* - [BP] GO:0006366: transcription from Pol II promoter \* - [BP] GO:0007165: signal transduction - [BP] GO:0007275: development \* | - cd00130: PAS domain - KOG3558: Hypoxia-inducible factor 1/Neuronal PAS domain protein NPAS1 [Signal transduction mechanisms, Transcription] - smart00353: helix loop helix domain |  |  |
| 8626 | TP73L | tumor protein p73-like | 3q27-q29 | - [MF] GO:0003700: transcription factor activity \* - [CC] GO:0005634: nucleus \* - [CC] GO:0005634: nucleus \* - [BP] GO:0006355: regulation of transcription, DNA-dependent \* - [BP] GO:0006355: regulation of transcription, DNA-dependent \* - [BP] GO:0006915: apoptosis - [BP] GO:0006917: induction of apoptosis - [MF] GO:0016563: transcriptional activator activity | - pfam00870: P53 - smart00454: Sterile alpha motif |  | - 103285 - 106260 - 603543 - 604292 - 605289 |
| 6591 | SNAI2 | snail homolog 2 (Drosophila) | 8q11 | - [BP] GO:0000122: negative regulation of transcription from Pol II promoter - [MF] GO:0003677: DNA binding \* - [CC] GO:0005634: nucleus \* - [BP] GO:0006355: regulation of transcription, DNA-dependent \* - [BP] GO:0007275: development \* - [BP] GO:0007499: ectoderm/mesoderm interaction - [MF] GO:0008270: zinc ion binding \* | - KOG2462: C2H2-type Zn-finger protein [Transcription] |  |  |
| 4609 | MYC | v-myc myelocytomatosis viral oncogene homolog (avian) | 8q24.12-q24.13 | - [MF] GO:0003700: transcription factor activity \* - [CC] GO:0005634: nucleus \* - [BP] GO:0006357: regulation of transcription from Pol II promoter \* - [BP] GO:0006879: iron ion homeostasis - [BP] GO:0007050: cell cycle arrest - [BP] GO:0008283: cell proliferation | - cd00083: Helix-loop-helix domain, found in specific DNA- binding proteins that act as transcription factors - pfam01056: Myc amino-terminal region - pfam02344: Myc leucine zipper domain | - MAPK signaling pathway - Wnt signaling pathway - TGF-beta signaling pathway - Jak-STAT signaling pathway | - 113970 |
| 23650 | TRIM29 | tripartite motif-containing 29 | 11q22-q23 | - [MF] GO:0003700: transcription factor activity \* - [CC] GO:0005622: intracellular \* - [BP] GO:0006366: transcription from Pol II promoter \* - [MF] GO:0008270: zinc ion binding \* | - smart00336: B-Box-type zinc finger |  |  |
| 6926 | TBX3 | T-box 3 (ulnar mammary syndrome) | 12q24.1 | - [BP] GO:0001501: skeletal development - [MF] GO:0003700: transcription factor activity \* - [MF] GO:0003702: RNA polymerase II transcription factor activity - [CC] GO:0005634: nucleus \* - [BP] GO:0006357: regulation of transcription from Pol II promoter \* - [BP] GO:0009653: morphogenesis \* | - cd00182: T-box DNA binding domain of the T-box family of transcriptional regulators |  | - 181450 |
| 5430 | POLR2A | polymerase (RNA) II (DNA directed) polypeptide A, 220kDa | 17p13.1 | - [MF] GO:0003677: DNA binding \* - [MF] GO:0003677: DNA binding \* - [MF] GO:0003899: DNA-directed RNA polymerase activity - [MF] GO:0005515: protein binding \* - [CC] GO:0005634: nucleus \* - [CC] GO:0005665: DNA-directed RNA polymerase II, core complex - [CC] GO:0005665: DNA-directed RNA polymerase II, core complex - [BP] GO:0006350: transcription - [BP] GO:0006351: transcription, DNA-dependent \* - [BP] GO:0006355: regulation of transcription, DNA-dependent \* - [BP] GO:0006366: transcription from Pol II promoter \* - [MF] GO:0016740: transferase activity | - COG0086: DNA-directed RNA polymerase, beta' subunit/160 kD subunit [Transcription] - KOG0260: RNA polymerase II, large subunit [Transcription] - pfam04983: RNA polymerase Rpb1, domain 3 - pfam04990: RNA polymerase Rpb1, domain 7 - pfam04992: RNA polymerase Rpb1, domain 6 - pfam04997: RNA polymerase Rpb1, domain 1 - pfam05000: RNA polymerase Rpb1, domain 4 - smart00663: RNA polymerase I subunit A N-terminus | - Purine metabolism - Pyrimidine metabolism - RNA polymerase |  |
| 8570 | KHSRP | KH-type splicing regulatory protein (FUSE binding protein 2) | 19p13.3 | - [BP] GO:0000398: nuclear mRNA splicing, via spliceosome - [MF] GO:0003677: DNA binding \* - [MF] GO:0003723: RNA binding - [CC] GO:0005634: nucleus \* - [BP] GO:0006355: regulation of transcription, DNA-dependent \* - [BP] GO:0006406: mRNA-nucleus export - [BP] GO:0006810: transport | - cd00105: K homology RNA-binding domain, binds single-stranded RNA, found in a wide variety of proteins including ribosomal proteins, transcription factors and post-transcriptional modifiers of mRNA - KOG1676: K-homology type RNA binding proteins [RNA processing and modification] |  |  |
| 10155 | TRIM28 | tripartite motif-containing 28 | 19q13.4 | - [CC] GO:0000151: ubiquitin ligase complex - [MF] GO:0003677: DNA binding \* - [MF] GO:0003700: transcription factor activity \* - [MF] GO:0003714: transcription corepressor activity - [MF] GO:0004842: ubiquitin-protein ligase activity - [CC] GO:0005634: nucleus \* - [BP] GO:0006355: regulation of transcription, DNA-dependent \* - [BP] GO:0006357: regulation of transcription from Pol II promoter \* - [MF] GO:0008270: zinc ion binding \* - [BP] GO:0016567: protein ubiquitination | - cd00021: B-Box-type zinc finger - pfam00628: PHD-finger - pfam00643: B-box zinc finger - smart00297: bromo domain - smart00502: B-Box C-terminal domain |  |  |
