# Supplementary material for: Ensemble attribute profile clustering: discovering and characterizing groups of genes with similar patterns of biological features
Source: BMC Bioinformatics. 2006 Mar 16;7:147. doi: 10.1186/1471-2105-7-147 (PMC1435935; doi:10.1186/1471-2105-7-147)
Supplement: Additional File 4 — Information on the 89 genes in the consensus clusters discovered and characterized for the MYOEPITHELIAL collection, along with their associated GO and CDD attributes. The formats are the same as for luminal-all.html and luminal-attrfreq.html. [file 1471-2105-7-147-S4.html]

Attribute breakdown

# Class 0 (48 LocusIDs)

| ID | Name | Count | Relative frequency |
| --- | --- | --- | --- |
| GO:0007582 | [BP] physiological process | 28 | 0.58 |
| GO:0005623 | [CC] cell | 27 | 0.56 |
| GO:0005622 | [CC] intracellular | 26 | 0.54 |
| GO:0005488 | [MF] binding | 22 | 0.46 |
| GO:0005737 | [CC] cytoplasm | 18 | 0.38 |
| GO:0007275 | [BP] development | 17 | 0.35 |
| GO:0008152 | [BP] metabolism | 17 | 0.35 |
| GO:0009987 | [BP] cellular process | 15 | 0.31 |
| GO:0009653 | [BP] morphogenesis | 14 | 0.29 |
| GO:0046872 | [MF] metal ion binding | 12 | 0.25 |
| GO:0043167 | [MF] ion binding | 12 | 0.25 |
| GO:0050875 | [BP] cellular physiological process | 12 | 0.25 |
| GO:0009887 | [BP] organogenesis | 12 | 0.25 |
| GO:0003824 | [MF] catalytic activity | 12 | 0.25 |
| GO:0008151 | [BP] cell growth and/or maintenance | 10 | 0.21 |
| GO:0005634 | [CC] nucleus | 7 | 0.15 |
| GO:0007398 | [BP] ectoderm development | 6 | 0.12 |
| GO:0050874 | [BP] organismal physiological process | 6 | 0.12 |
| GO:0016491 | [MF] oxidoreductase activity | 6 | 0.12 |
| GO:0005576 | [CC] extracellular | 6 | 0.12 |
| GO:0008544 | [BP] epidermis development | 6 | 0.12 |
| GO:0005215 | [MF] transporter activity | 5 | 0.10 |
| GO:0005578 | [CC] extracellular matrix | 4 | 0.08 |
| GO:0004866 | [MF] endopeptidase inhibitor activity | 4 | 0.08 |
| GO:0008283 | [BP] cell proliferation | 4 | 0.08 |
| GO:0016740 | [MF] transferase activity | 4 | 0.08 |
| GO:0005198 | [MF] structural molecule activity | 4 | 0.08 |
| GO:0006810 | [BP] transport | 4 | 0.08 |
| GO:0007154 | [BP] cell communication | 4 | 0.08 |
| GO:0006092 | [BP] main pathways of carbohydrate metabolism | 3 | 0.06 |
| GO:0046914 | [MF] transition metal ion binding | 3 | 0.06 |
| GO:0006007 | [BP] glucose catabolism | 3 | 0.06 |
| GO:0008270 | [MF] zinc ion binding | 3 | 0.06 |
| GO:0016021 | [CC] integral to membrane | 3 | 0.06 |
| GO:0005489 | [MF] electron transporter activity | 3 | 0.06 |
| GO:0005507 | [MF] copper ion binding | 3 | 0.06 |
| GO:0005515 | [MF] protein binding | 3 | 0.06 |
| GO:0001501 | [BP] skeletal development | 3 | 0.06 |
| GO:0005509 | [MF] calcium ion binding | 3 | 0.06 |
| GO:0003676 | [MF] nucleic acid binding | 3 | 0.06 |
| GO:0005739 | [CC] mitochondrion | 3 | 0.06 |
| GO:0000287 | [MF] magnesium ion binding | 3 | 0.06 |
| GO:0007399 | [BP] neurogenesis | 3 | 0.06 |
| GO:0007165 | [BP] signal transduction | 3 | 0.06 |
| GO:0004871 | [MF] signal transducer activity | 3 | 0.06 |
| GO:0006096 | [BP] glycolysis | 3 | 0.06 |
| GO:0016020 | [CC] membrane | 3 | 0.06 |
| GO:0003723 | [MF] RNA binding | 2 | 0.04 |
| GO:0005525 | [MF] GTP binding | 2 | 0.04 |
| GO:0019001 | [MF] guanyl nucleotide binding | 2 | 0.04 |
| GO:0030154 | [BP] cell differentiation | 2 | 0.04 |
| GO:0016787 | [MF] hydrolase activity | 2 | 0.04 |
| GO:0005102 | [MF] receptor binding | 2 | 0.04 |
| GO:0005783 | [CC] endoplasmic reticulum | 2 | 0.04 |
| GO:0005615 | [CC] extracellular space | 2 | 0.04 |
| cd00083 | [CDD] Helix-loop-helix domain, found in specific DNA- binding proteins that act as transcription factors | 2 | 0.04 |
| GO:0005518 | [MF] collagen binding | 2 | 0.04 |

# Class 1 (17 LocusIDs)

| ID | Name | Count | Relative frequency |
| --- | --- | --- | --- |
| GO:0005623 | [CC] cell | 17 | 1.00 |
| GO:0016020 | [CC] membrane | 16 | 0.94 |
| GO:0016021 | [CC] integral to membrane | 15 | 0.88 |
| GO:0009987 | [BP] cellular process | 15 | 0.88 |
| GO:0007154 | [BP] cell communication | 12 | 0.71 |
| GO:0005488 | [MF] binding | 11 | 0.65 |
| GO:0007582 | [BP] physiological process | 10 | 0.59 |
| GO:0005886 | [CC] plasma membrane | 10 | 0.59 |
| GO:0007155 | [BP] cell adhesion | 10 | 0.59 |
| GO:0050875 | [BP] cellular physiological process | 9 | 0.53 |
| GO:0005887 | [CC] integral to plasma membrane | 9 | 0.53 |
| GO:0005515 | [MF] protein binding | 8 | 0.47 |
| GO:0008151 | [BP] cell growth and/or maintenance | 8 | 0.47 |
| GO:0016337 | [BP] cell-cell adhesion | 7 | 0.41 |
| GO:0004871 | [MF] signal transducer activity | 7 | 0.41 |
| GO:0004872 | [MF] receptor activity | 5 | 0.29 |
| GO:0005576 | [CC] extracellular | 5 | 0.29 |
| GO:0046872 | [MF] metal ion binding | 4 | 0.24 |
| GO:0043167 | [MF] ion binding | 4 | 0.24 |
| GO:0007275 | [BP] development | 4 | 0.24 |
| GO:0005509 | [MF] calcium ion binding | 4 | 0.24 |
| GO:0005624 | [CC] membrane fraction | 3 | 0.18 |
| GO:0005215 | [MF] transporter activity | 3 | 0.18 |
| cd00054 | [CDD] Calcium-binding EGF-like domain, present in a large number of membrane-bound and extracellular (mostly animal) proteins. Many of these proteins require calcium for their biological function and calcium-binding sites have been found to be located at the N- | 3 | 0.18 |
| GO:0000267 | [CC] cell fraction | 3 | 0.18 |
| GO:0005615 | [CC] extracellular space | 3 | 0.18 |
| GO:0005198 | [MF] structural molecule activity | 3 | 0.18 |
| KOG4289 | [CDD] Cadherin EGF LAG seven-pass G-type receptor [Signal transduction mechanisms] | 3 | 0.18 |
| GO:0006810 | [BP] transport | 3 | 0.18 |
| cd00031 | [CDD] Cadherin repeat domain | 3 | 0.18 |
| GO:0007156 | [BP] homophilic cell adhesion | 3 | 0.18 |
| GO:0007160 | [BP] cell-matrix adhesion | 3 | 0.18 |
| GO:0007165 | [BP] signal transduction | 3 | 0.18 |
| GO:0007166 | [BP] cell surface receptor linked signal transduction | 3 | 0.18 |
| GO:0012501 | [BP] programmed cell death | 2 | 0.12 |
| GO:0005578 | [CC] extracellular matrix | 2 | 0.12 |
| GO:0008283 | [BP] cell proliferation | 2 | 0.12 |
| GO:0009653 | [BP] morphogenesis | 2 | 0.12 |
| GO:0005102 | [MF] receptor binding | 2 | 0.12 |
| GO:0009887 | [BP] organogenesis | 2 | 0.12 |
| GO:0007399 | [BP] neurogenesis | 2 | 0.12 |
| GO:0006915 | [BP] apoptosis | 2 | 0.12 |
| GO:0003824 | [MF] catalytic activity | 2 | 0.12 |
| GO:0005622 | [CC] intracellular | 2 | 0.12 |
| GO:0008083 | [MF] growth factor activity | 2 | 0.12 |

# Class 2 (15 LocusIDs)

| ID | Name | Count | Relative frequency |
| --- | --- | --- | --- |
| GO:0007582 | [BP] physiological process | 15 | 1.00 |
| GO:0019538 | [BP] protein metabolism | 15 | 1.00 |
| GO:0008152 | [BP] metabolism | 15 | 1.00 |
| GO:0003824 | [MF] catalytic activity | 12 | 0.80 |
| GO:0005488 | [MF] binding | 10 | 0.67 |
| GO:0016787 | [MF] hydrolase activity | 9 | 0.60 |
| GO:0009987 | [BP] cellular process | 8 | 0.53 |
| GO:0050875 | [BP] cellular physiological process | 7 | 0.47 |
| GO:0006508 | [BP] proteolysis and peptidolysis | 7 | 0.47 |
| GO:0030163 | [BP] protein catabolism | 7 | 0.47 |
| GO:0005576 | [CC] extracellular | 7 | 0.47 |
| GO:0008151 | [BP] cell growth and/or maintenance | 7 | 0.47 |
| GO:0005623 | [CC] cell | 7 | 0.47 |
| GO:0008283 | [BP] cell proliferation | 6 | 0.40 |
| GO:0006464 | [BP] protein modification | 6 | 0.40 |
| GO:0005622 | [CC] intracellular | 6 | 0.40 |
| GO:0007049 | [BP] cell cycle | 5 | 0.33 |
| GO:0000074 | [BP] regulation of cell cycle | 5 | 0.33 |
| GO:0005524 | [MF] ATP binding | 5 | 0.33 |
| GO:0030554 | [MF] adenyl nucleotide binding | 5 | 0.33 |
| GO:0007154 | [BP] cell communication | 5 | 0.33 |
| GO:0007165 | [BP] signal transduction | 5 | 0.33 |
| GO:0005737 | [CC] cytoplasm | 5 | 0.33 |
| GO:0006468 | [BP] protein amino acid phosphorylation | 4 | 0.27 |
| GO:0005515 | [MF] protein binding | 4 | 0.27 |
| GO:0016310 | [BP] phosphorylation | 4 | 0.27 |
| GO:0004295 | [MF] trypsin activity | 3 | 0.20 |
| GO:0004252 | [MF] serine-type endopeptidase activity | 3 | 0.20 |
| GO:0016740 | [MF] transferase activity | 3 | 0.20 |
| GO:0005615 | [CC] extracellular space | 3 | 0.20 |
| GO:0050874 | [BP] organismal physiological process | 3 | 0.20 |
| GO:0008284 | [BP] positive regulation of cell proliferation | 2 | 0.13 |
| GO:0046872 | [MF] metal ion binding | 2 | 0.13 |
| GO:0005578 | [CC] extracellular matrix | 2 | 0.13 |
| GO:0016021 | [CC] integral to membrane | 2 | 0.13 |
| GO:0043167 | [MF] ion binding | 2 | 0.13 |
| GO:0007275 | [BP] development | 2 | 0.13 |
| GO:0042127 | [BP] regulation of cell proliferation | 2 | 0.13 |
| GO:0005509 | [MF] calcium ion binding | 2 | 0.13 |
| GO:0004871 | [MF] signal transducer activity | 2 | 0.13 |
| GO:0007166 | [BP] cell surface receptor linked signal transduction | 2 | 0.13 |
| GO:0016020 | [CC] membrane | 2 | 0.13 |

# Class 3 (9 LocusIDs)

| ID | Name | Count | Relative frequency |
| --- | --- | --- | --- |
| GO:0007582 | [BP] physiological process | 9 | 1.00 |
| GO:0003676 | [MF] nucleic acid binding | 9 | 1.00 |
| GO:0006351 | [BP] transcription, DNA-dependent | 9 | 1.00 |
| GO:0003677 | [MF] DNA binding | 9 | 1.00 |
| GO:0005488 | [MF] binding | 9 | 1.00 |
| GO:0005622 | [CC] intracellular | 9 | 1.00 |
| GO:0008152 | [BP] metabolism | 9 | 1.00 |
| GO:0005623 | [CC] cell | 9 | 1.00 |
| GO:0005634 | [CC] nucleus | 8 | 0.89 |
| GO:0006355 | [BP] regulation of transcription, DNA-dependent | 8 | 0.89 |
| GO:0045449 | [BP] regulation of transcription | 8 | 0.89 |
| GO:0006366 | [BP] transcription from Pol II promoter | 7 | 0.78 |
| GO:0003700 | [MF] transcription factor activity | 6 | 0.67 |
| GO:0030528 | [MF] transcription regulator activity | 6 | 0.67 |
| GO:0006357 | [BP] regulation of transcription from Pol II promoter | 4 | 0.44 |
| GO:0009987 | [BP] cellular process | 4 | 0.44 |
| GO:0046872 | [MF] metal ion binding | 3 | 0.33 |
| GO:0046914 | [MF] transition metal ion binding | 3 | 0.33 |
| GO:0008270 | [MF] zinc ion binding | 3 | 0.33 |
| GO:0043167 | [MF] ion binding | 3 | 0.33 |
| GO:0050875 | [BP] cellular physiological process | 3 | 0.33 |
| GO:0007275 | [BP] development | 3 | 0.33 |
| GO:0009653 | [BP] morphogenesis | 3 | 0.33 |
| GO:0005515 | [MF] protein binding | 3 | 0.33 |
| GO:0009887 | [BP] organogenesis | 3 | 0.33 |
| GO:0003712 | [MF] transcription cofactor activity | 2 | 0.22 |
| GO:0003824 | [MF] catalytic activity | 2 | 0.22 |
| GO:0008151 | [BP] cell growth and/or maintenance | 2 | 0.22 |

# Summary

| ID | Name | 0 | 1 | 2 | 3 |
| --- | --- | --- | --- | --- | --- |
| GO:0007582 | physiological process | 28 | 10 | 15 | 9 |
| GO:0005623 | cell | 27 | 17 | 7 | 9 |
| GO:0005488 | binding | 22 | 11 | 10 | 9 |
| GO:0005622 | intracellular | 26 | 2 | 6 | 9 |
| GO:0009987 | cellular process | 15 | 15 | 8 | 4 |
| GO:0050875 | cellular physiological process | 12 | 9 | 7 | 3 |
| GO:0003824 | catalytic activity | 12 | 2 | 12 | 2 |
| GO:0008151 | cell growth and/or maintenance | 10 | 8 | 7 | 2 |
| GO:0007275 | development | 17 | 4 | 2 | 3 |
| GO:0007154 | cell communication | 4 | 12 | 5 | 1 |
| GO:0043167 | ion binding | 12 | 4 | 2 | 3 |
| GO:0046872 | metal ion binding | 12 | 4 | 2 | 3 |
| GO:0009653 | morphogenesis | 14 | 2 | 1 | 3 |
| GO:0005515 | protein binding | 3 | 8 | 4 | 3 |
| GO:0005634 | nucleus | 7 | 1 | 1 | 8 |
| GO:0008283 | cell proliferation | 4 | 2 | 6 | 1 |
| GO:0004871 | signal transducer activity | 3 | 7 | 2 | 1 |
| GO:0007165 | signal transduction | 3 | 3 | 5 | 1 |
| GO:0006810 | transport | 4 | 3 | 1 | 1 |
| GO:0006915 | apoptosis | 1 | 2 | 1 | 1 |
| GO:0012501 | programmed cell death | 1 | 2 | 1 | 1 |
| GO:0008152 | metabolism | 17 | 0 | 15 | 9 |
| GO:0005737 | cytoplasm | 18 | 1 | 5 | 0 |
| GO:0016020 | membrane | 3 | 16 | 2 | 0 |
| GO:0016021 | integral to membrane | 3 | 15 | 2 | 0 |
| GO:0005576 | extracellular | 6 | 5 | 7 | 0 |
| GO:0009887 | organogenesis | 12 | 2 | 0 | 3 |
| GO:0019538 | protein metabolism | 1 | 0 | 15 | 1 |
| GO:0003676 | nucleic acid binding | 3 | 0 | 1 | 9 |
| GO:0016787 | hydrolase activity | 2 | 1 | 9 | 0 |
| GO:0050874 | organismal physiological process | 6 | 1 | 3 | 0 |
| GO:0005509 | calcium ion binding | 3 | 4 | 2 | 0 |
| GO:0005578 | extracellular matrix | 4 | 2 | 2 | 0 |
| GO:0016740 | transferase activity | 4 | 0 | 3 | 1 |
| GO:0005615 | extracellular space | 2 | 3 | 3 | 0 |
| GO:0008270 | zinc ion binding | 3 | 0 | 1 | 3 |
| GO:0046914 | transition metal ion binding | 3 | 0 | 1 | 3 |
| GO:0007166 | cell surface receptor linked signal transduction | 1 | 3 | 2 | 0 |
| GO:0003723 | RNA binding | 2 | 0 | 1 | 1 |
| GO:0042127 | regulation of cell proliferation | 1 | 1 | 2 | 0 |
| GO:0003677 | DNA binding | 0 | 0 | 1 | 9 |
| GO:0005215 | transporter activity | 5 | 3 | 0 | 0 |
| GO:0005198 | structural molecule activity | 4 | 3 | 0 | 0 |
| GO:0030528 | transcription regulator activity | 1 | 0 | 0 | 6 |
| GO:0006464 | protein modification | 0 | 0 | 6 | 1 |
| GO:0004872 | receptor activity | 0 | 5 | 1 | 0 |
| GO:0000074 | regulation of cell cycle | 0 | 0 | 5 | 1 |
| GO:0007049 | cell cycle | 0 | 0 | 5 | 1 |
| GO:0004866 | endopeptidase inhibitor activity | 4 | 0 | 1 | 0 |
| GO:0007399 | neurogenesis | 3 | 2 | 0 | 0 |
| GO:0001501 | skeletal development | 3 | 0 | 0 | 1 |
| GO:0005102 | receptor binding | 2 | 2 | 0 | 0 |
| GO:0000267 | cell fraction | 0 | 3 | 1 | 0 |
| GO:0005518 | collagen binding | 2 | 1 | 0 | 0 |
| GO:0005783 | endoplasmic reticulum | 2 | 1 | 0 | 0 |
| GO:0030154 | cell differentiation | 2 | 1 | 0 | 0 |
| GO:0019001 | guanyl nucleotide binding | 2 | 0 | 1 | 0 |
| GO:0005525 | GTP binding | 2 | 0 | 1 | 0 |
| cd00083 | Helix-loop-helix domain, found in specific DNA- binding proteins that act as transcription factors | 2 | 0 | 0 | 1 |
| GO:0008083 | growth factor activity | 1 | 2 | 0 | 0 |
| GO:0008284 | positive regulation of cell proliferation | 1 | 0 | 2 | 0 |
| GO:0003712 | transcription cofactor activity | 1 | 0 | 0 | 2 |
| GO:0007155 | cell adhesion | 0 | 10 | 0 | 0 |
| GO:0005886 | plasma membrane | 0 | 10 | 0 | 0 |
| GO:0005887 | integral to plasma membrane | 0 | 9 | 0 | 0 |
| GO:0006351 | transcription, DNA-dependent | 0 | 0 | 0 | 9 |
| GO:0045449 | regulation of transcription | 0 | 0 | 0 | 8 |
| GO:0006355 | regulation of transcription, DNA-dependent | 0 | 0 | 0 | 8 |
| GO:0016337 | cell-cell adhesion | 0 | 7 | 0 | 0 |
| GO:0030163 | protein catabolism | 0 | 0 | 7 | 0 |
| GO:0006508 | proteolysis and peptidolysis | 0 | 0 | 7 | 0 |
| GO:0006366 | transcription from Pol II promoter | 0 | 0 | 0 | 7 |
| GO:0008544 | epidermis development | 6 | 0 | 0 | 0 |
| GO:0016491 | oxidoreductase activity | 6 | 0 | 0 | 0 |
| GO:0007398 | ectoderm development | 6 | 0 | 0 | 0 |
| GO:0003700 | transcription factor activity | 0 | 0 | 0 | 6 |
| GO:0030554 | adenyl nucleotide binding | 0 | 0 | 5 | 0 |
| GO:0005524 | ATP binding | 0 | 0 | 5 | 0 |
| GO:0016310 | phosphorylation | 0 | 0 | 4 | 0 |
| GO:0006468 | protein amino acid phosphorylation | 0 | 0 | 4 | 0 |
| GO:0006357 | regulation of transcription from Pol II promoter | 0 | 0 | 0 | 4 |
| GO:0006096 | glycolysis | 3 | 0 | 0 | 0 |
| GO:0000287 | magnesium ion binding | 3 | 0 | 0 | 0 |
| GO:0005739 | mitochondrion | 3 | 0 | 0 | 0 |
| GO:0005507 | copper ion binding | 3 | 0 | 0 | 0 |
| GO:0005489 | electron transporter activity | 3 | 0 | 0 | 0 |
| GO:0006007 | glucose catabolism | 3 | 0 | 0 | 0 |
| GO:0006092 | main pathways of carbohydrate metabolism | 3 | 0 | 0 | 0 |
| GO:0007160 | cell-matrix adhesion | 0 | 3 | 0 | 0 |
| GO:0007156 | homophilic cell adhesion | 0 | 3 | 0 | 0 |
| cd00031 | Cadherin repeat domain | 0 | 3 | 0 | 0 |
| KOG4289 | Cadherin EGF LAG seven-pass G-type receptor [Signal transduction mechanisms] | 0 | 3 | 0 | 0 |
| cd00054 | Calcium-binding EGF-like domain, present in a large number of membrane-bound and extracellular (mostly animal) proteins. Many of these proteins require calcium for their biological function and calcium-binding sites have been found to be located at the N- | 0 | 3 | 0 | 0 |
| GO:0005624 | membrane fraction | 0 | 3 | 0 | 0 |
| GO:0004252 | serine-type endopeptidase activity | 0 | 0 | 3 | 0 |
| GO:0004295 | trypsin activity | 0 | 0 | 3 | 0 |
