# Supplementary material for: Ensemble attribute profile clustering: discovering and characterizing groups of genes with similar patterns of biological features
Source: BMC Bioinformatics. 2006 Mar 16;7:147. doi: 10.1186/1471-2105-7-147 (PMC1435935; doi:10.1186/1471-2105-7-147)
Supplement: Additional File 5 — Information on the chromosomal locations of the luminal epithelial (brown) and myoepithelial (green) genes. For each gene, the "LocusID" and "Symbol" fields are taken from the July 2004 release of LocusLink. The "Cytoband", "Start" and "End" fields are taken from the May 2004 UCSC assembly of the human genome. Genes are ordered by chromosomal coordinates (p arm followed by q arm). The "Overall" column indicates the distance in kilobases between the adjacent transcription end and start coordinates of the two closest genes from the union of the LUMINAL and MYOEPITHELIAL collections, as well as the number of RefSeqs found in this interval. "pter" and "qter" indicate the number of genes from a telomere to the closest gene in either collection. [file 1471-2105-7-147-S5.html]

Interlopers


# Chromosome 1 (12 loci)

LuminalMyoepithelial | Luminal | Myoepithelial | LocusID | Symbol | Cytoband | Start | End || pter: 227 |  |  | 3399 | ID3 | p36.12 | 23,629,761 | 23,631,591 |
| 54  3242   16.66 | 54  3242   16.66 |
| 2810 | SFN | p36.11 | 26,873,774 | 26,875,089 |
| 228  18771   12.15 | 228  18771   12.15 |
| 5052 | PRDX1 | p34.1 | 45,645,800 | 45,656,702 |
| 73  9371   7.79 | 497  104160   4.77 |
| 1718 | DHCR24 | p32.3 | 55,027,328 | 55,064,912 |
| 374  92852   4.03 | 374  92852   4.03 |
| 10500 | SEMA6C | q21.2 | 147,917,234 | 147,932,177 |
| 48  1885   25.47 | 93  3715   25.03 |
| 6699 | SPRR1B | q21.3 | 149,816,753 | 149,818,449 |
| 9  425   21.18 | 9  425   21.18 |
| 6278 | S100A7 | q21.3 | 150,243,293 | 150,246,210 |
| 4  100   39.82 | 4  100   39.82 |
| 6273 | S100A2 | q21.3 | 150,346,659 | 150,351,379 |
| 29  1296   22.37 | 232  16072   14.43 |
| 4582 | MUC1 | q22 | 151,647,667 | 151,975,753 |
| 176  13832   12.72 | 176  13832   12.72 |
| 481 | ATP1B1 | q24.2 | 165,807,604 | 165,833,618 |
| 6  590   10.17 | 65  11022   5.90 |
| 6401 | SELE | q24.2 | 166,423,439 | 166,434,837 |
| 58  10421   5.57 |  |
| 5768 | QSCN6 | q25.2 | 176,855,624 | 176,898,824 |
| qter: 372 |  |

# Chromosome 2 (9 loci)

Overall | jones-supp-luminal | jones-supp-myoepithelial | Locus ID | Symbol | Cytoband | Start | End || pter: 21 |  |  | 3398 | ID2 | p25.1 | 8,772,710 | 8,775,179 |
| 183  37661   4.86 | 183  37661   4.86 |
| 2034 | EPAS1 | p21 | 46,436,213 | 46,525,486 |
| 40  14064   2.84 | 128  27812   4.60 |
| 53335 | BCL11A | p16.1 | 60,589,952 | 60,692,284 |
| 87  13645   6.38 | 193  36866   5.24 |
| 10797 | MTHFD2 | p13.1 | 74,337,366 | 74,354,067 |
| 105  23204   4.53 | 245  56271   4.35 |
| 57730 | KIAA1641 | q11.2 | 97,557,788 | 97,586,230 |
| 139  33039   4.21 |  |
| 55627 | FLJ20297 | q21.1 | 130,625,211 | 130,655,396 |
| 127  42462   2.99 | 127  42462   2.99 |
| 3655 | ITGA6 | q31.1 | 173,117,876 | 173,196,509 |
| 64  16526   3.87 | 64  16526   3.87 |
| 1290 | COL5A2 | q32.2 | 189,722,746 | 189,869,994 |
| 291  51225   5.68 | 291  51225   5.68 |
| 2817 | GPC1 | q37.3 | 241,095,104 | 241,127,483 |
| qter: 25 |  |

# Chromosome 3 (11 loci)

Overall | jones-supp-luminal | jones-supp-myoepithelial | Locus ID | Symbol | Cytoband | Start | End || pter: 77 |  |  | 9467 | SH3BP5 | p25.1 | 15,271,367 | 15,348,892 |
| 102  27268   3.74 | 102  27268   3.74 |
| 4820 | NKTR | p22.1 | 42,617,109 | 42,665,031 |
| 125  7616   16.41 | 125  7616   16.41 |
| 7869 | SEMA3B | p21.31 | 50,281,441 | 50,289,575 |
| 118  18927   6.23 | 118  18927   6.23 |
| 10550 | JWA | p14.1 | 69,216,799 | 69,237,907 |
| 132  54289   2.43 | 168  59656   2.82 |
| 1475 | CSTA | q21.1 | 123,526,772 | 123,543,201 |
| 13  1271   10.23 | 13  1271   10.23 |
| 4638 | MYLK | q21.1 | 124,813,834 | 125,085,839 |
| 21  3808   5.51 | 112  17992   6.22 |
| 11343 | MGLL | q21.3 | 128,893,656 | 129,024,423 |
| 90  14054   6.40 | 158  30873   5.12 |
| 483 | ATP1B3 | q23 | 143,078,167 | 143,128,080 |
| 67  16770   4.00 | 189  47704   3.96 |
| 5918 | RARRES1 | q25.32 | 159,897,600 | 159,932,977 |
| 121  30899   3.92 | 164  38285   4.28 |
| 8626 | TP73L | q28 | 190,831,917 | 191,097,764 |
| 42  7121   5.90 |  |
| 4241 | MFI2 | q29 | 198,218,465 | 198,244,952 |
| qter: 8 |  |

# Chromosome 4 (2 loci)

Overall | jones-supp-luminal | jones-supp-myoepithelial | Locus ID | Symbol | Cytoband | Start | End || pter: 22 |  |  | 10296 | MAEA | p16.3 | 1,273,542 | 1,323,721 |
| 258  74352   3.47 | 258  74352   3.47 |
| 374 | AREG | q13.3 | 75,675,887 | 75,685,760 |
| qter: 369 |  |

# Chromosome 5 (5 loci)

Overall | jones-supp-luminal | jones-supp-myoepithelial | Locus ID | Symbol | Cytoband | Start | End || pter: 297 |  |  | 2745 | GLRX | q15 | 95,175,430 | 95,184,137 |
| 236  45606   5.17 |  |
| 26025 | PCDHGA12 | q31.3 | 140,790,341 | 140,872,730 |
| 41  7983   5.14 | 41  7983   5.14 |
| 1452 | CSNK1A1 | q32 | 148,855,741 | 148,910,850 |
| 28  1953   14.34 | 28  1953   14.34 |
| 2196 | FAT2 | q33.1 | 150,863,846 | 150,928,698 |
| 0  93   0.00 | 0  93   0.00 |
| 6678 | SPARC | q33.1 | 151,021,203 | 151,046,710 |
| qter: 160 |  |

# Chromosome 7 (4 loci)

Overall | jones-supp-luminal | jones-supp-myoepithelial | Locus ID | Symbol | Cytoband | Start | End || pter: 107 |  |  | 8935 | SCAP2 | p15.2 | 26,479,928 | 26,677,581 |
| 173  46012   3.76 | 173  46012   3.76 |
| 1364 | CLDN4 | q11.23 | 72,689,843 | 72,691,665 |
| 246  43067   5.71 |  |
| 857 | CAV1 | q31.2 | 115,758,789 | 115,795,181 |
| 125  26148   4.78 | 125  26148   4.78 |
| 5644 | PRSS1 | q34 | 141,943,618 | 141,947,210 |
| qter: 95 |  |

# Chromosome 8 (6 loci)

Overall | jones-supp-luminal | jones-supp-myoepithelial | Locus ID | Symbol | Cytoband | Start | End || pter: 199 |  |  | 5327 | PLAT | p11.21 | 42,151,911 | 42,184,351 |
| 19  7808   2.43 | 19  7808   2.43 |
| 6591 | SNAI2 | q11.21 | 49,992,795 | 49,996,541 |
| 94  32359   2.90 | 94  32359   2.90 |
| 2171 | FABP5 | q21.13 | 82,355,339 | 82,359,563 |
| 90  22021   4.09 | 90  22021   4.09 |
| 8323 | FZD6 | q22.3 | 104,380,275 | 104,414,268 |
| 66  24403   2.70 | 66  24403   2.70 |
| 4609 | MYC | q24.21 | 128,817,685 | 128,822,853 |
| 13  5496   2.37 | 13  5496   2.37 |
| 10397 | NDRG1 | q24.22 | 134,318,595 | 134,378,680 |
| qter: 93 |  |

# Chromosome 9 (3 loci)

Overall | jones-supp-luminal | jones-supp-myoepithelial | Locus ID | Symbol | Cytoband | Start | End || pter: 239 |  |  | 1514 | CTSL | q21.33 | 87,570,533 | 87,575,862 |
| 226  40415   5.59 | 307  45682   6.72 |
| 3934 | LCN2 | q34.11 | 127,991,271 | 127,995,288 |
| 80  5263   15.20 |  |
| 6836 | SURF4 | q34.2 | 133,257,894 | 133,272,524 |
| qter: 84 |  |

# Chromosome 10 (7 loci)

Overall | jones-supp-luminal | jones-supp-myoepithelial | Locus ID | Symbol | Cytoband | Start | End || pter: 14 |  |  | 1646 | AKR1C2 | p15.1 | 5,021,965 | 5,050,207 |
| 21  6050   3.47 | 122  38151   3.20 |
| 10659 | CUGBP2 | p14 | 11,100,075 | 11,416,358 |
| 100  31785   3.15 | 208  60896   3.42 |
| 3185 | HNRPF | q11.21 | 43,201,070 | 43,224,620 |
| 107  29087   3.68 | 352  62780   5.61 |
| 5092 | PCBD | q22.1 | 72,312,045 | 72,318,547 |
| 56  9586   5.84 | 56  9586   5.84 |
| 311 | ANXA11 | q22.3 | 81,904,859 | 81,955,308 |
| 187  24049   7.78 |  |
| 9446 | GSTO1 | q25.1 | 106,004,667 | 106,017,199 |
| 68  18194   3.74 | 68  18194   3.74 |
| 5654 | PRSS11 | q26.13 | 124,211,110 | 124,264,413 |
| qter: 61 |  |

# Chromosome 11 (12 loci)

Overall | jones-supp-luminal | jones-supp-myoepithelial | Locus ID | Symbol | Cytoband | Start | End || pter: 35 |  |  | 977 | CD151 | p15.5 | 822,985 | 828,834 |
| 130  11112   11.70 | 423  62232   6.80 |
| 27122 | DKK3 | p15.3 | 11,941,228 | 11,987,493 |
| 104  23129   4.50 | 104  23129   4.50 |
| 960 | CD44 | p13 | 35,116,992 | 35,210,524 |
| 38  11445   3.32 | 38  11445   3.32 |
| 392 | ARHGAP1 | p11.2 | 46,655,209 | 46,678,696 |
| 148  16382   9.03 | 289  20429   14.15 |
| 5920 | RARRES3 | q12.3 | 63,060,855 | 63,070,505 |
| 1  28   35.31 | 1  28   35.31 |
| 11145 | HRASLS3 | q12.3–q13.1 | 63,098,824 | 63,138,469 |
| 84  2398   35.04 | 84  2398   35.04 |
| 1474 | CST6 | q13.1 | 65,536,037 | 65,537,551 |
| 53  1570   33.75 |  |
| 2950 | GSTP1 | q13.2 | 67,107,861 | 67,110,699 |
| 84  7840   10.71 | 84  7840   10.71 |
| 871 | SERPINH1 | q13.5 | 74,950,817 | 74,961,492 |
| 51  12705   4.01 | 51  12705   4.01 |
| 1075 | CTSC | q14.2 | 87,666,407 | 87,710,586 |
| 47  14501   3.24 | 47  14501   3.24 |
| 4314 | MMP3 | q22.2 | 102,211,742 | 102,219,550 |
| 133  17268   7.70 | 133  17268   7.70 |
| 23650 | TRIM29 | q23.3 | 119,487,204 | 119,514,073 |
| qter: 86 |  |

# Chromosome 12 (10 loci)

Overall | jones-supp-luminal | jones-supp-myoepithelial | Locus ID | Symbol | Cytoband | Start | End || pter: 56 |  |  | 2597 | GAPD | p13.31 | 6,513,944 | 6,517,797 |
| 184  21484   8.56 | 184  21484   8.56 |
| 5744 | PTHLH | p11.22 | 28,002,283 | 28,016,183 |
| 142  23339   6.08 | 142  23339   6.08 |
| 3848 | KRT1 | q13.13 | 51,354,718 | 51,360,446 |
| 77  3400   22.65 | 115  4549   25.28 |
| 2065 | ERBB3 | q13.2 | 54,760,153 | 54,782,854 |
| 37  1127   32.83 |  |
| 6472 | SHMT2 | q13.3 | 55,909,818 | 55,914,981 |
| 64  12351   5.18 | 64  12351   5.18 |
| 10576 | CCT2 | q15 | 68,265,511 | 68,281,617 |
| 29  7473   3.88 | 29  7473   3.88 |
| 1466 | CSRP2 | q21.2 | 75,754,963 | 75,775,267 |
| 23  12469   1.84 | 23  12469   1.84 |
| 1848 | DUSP6 | q21.33 | 88,244,306 | 88,248,764 |
| 163  25322   6.44 | 163  25322   6.44 |
| 6926 | TBX3 | q24.21 | 113,570,778 | 113,584,689 |
| 106  16297   6.50 | 106  16297   6.50 |
| 5901 | RAN | q24.33 | 129,881,447 | 129,886,243 |
| qter: 22 |  |

# Chromosome 13 (1 locus)

Overall | jones-supp-luminal | jones-supp-myoepithelial | Locus ID | Symbol | Cytoband | Start | End || pter: 43 |  |  | 2971 | GTF3A | q12.2 | 26,896,680 | 26,907,823 |
| qter: 239 |  |

# Chromosome 14 (5 loci)

Overall | jones-supp-luminal | jones-supp-myoepithelial | Locus ID | Symbol | Cytoband | Start | End || pter: 73 |  |  | 5720 | PSME1 | q11.2 | 23,675,217 | 23,678,015 |
| 125  30988   4.03 | 125  30988   4.03 |
| 3958 | LGALS3 | q22.3 | 54,665,757 | 54,681,881 |
| 70  13642   5.13 | 70  13642   5.13 |
| 677 | ZFP36L1 | q24.1 | 68,324,127 | 68,329,538 |
| 49  6210   7.89 | 195  34333   5.68 |
| 8892 | EIF2B2 | q24.3 | 74,539,400 | 74,546,045 |
| 145  28116   5.16 |  |
| 7127 | TNFAIP2 | q32.32 | 102,662,416 | 102,673,529 |
| qter: 34 |  |

# Chromosome 15 (2 loci)

Overall | jones-supp-luminal | jones-supp-myoepithelial | Locus ID | Symbol | Cytoband | Start | End || pter: 303 |  |  | 5315 | PKM2 | q23 | 70,278,423 | 70,310,738 |
| 172  26700   6.44 | 172  26700   6.44 |
| 3480 | IGF1R | q26.3 | 97,010,287 | 97,319,034 |
| qter: 19 |  |

# Chromosome 16 (11 loci)

Overall | jones-supp-luminal | jones-supp-myoepithelial | Locus ID | Symbol | Cytoband | Start | End || pter: 127 |  |  | 9235 | NK4 | p13.3 | 3,055,313 | 3,059,549 |
| 38  1783   21.32 | 38  1783   21.32 |
| 29855 | UBN1 | p13.3 | 4,842,216 | 4,872,364 |
| 200  26178   7.64 | 200  26178   7.64 |
| 5652 | PRSS8 | p11.2 | 31,050,256 | 31,054,652 |
| 65  24126   2.69 | 87  24909   3.49 |
| 4504 | MT3 | q12.2 | 55,180,767 | 55,182,500 |
| 2  42   48.11 | 2  42   48.11 |
| 4499 | MT1K | q12.2 | 55,224,072 | 55,225,392 |
| 2  18   111.61 | 2  18   111.61 |
| 4490 | MT1B | q12.2 | 55,243,311 | 55,244,616 |
| 0  5   0.00 | 0  5   0.00 |
| 4494 | MT1F | q12.2 | 55,249,355 | 55,250,713 |
| 0  7   0.00 | 0  7   0.00 |
| 4495 | MT1G | q12.2 | 55,258,153 | 55,259,478 |
| 1  14   69.36 | 1  14   69.36 |
| 4501 | MT1X | q12.2 | 55,273,895 | 55,275,608 |
| 11  688   15.98 | 196  25942   7.56 |
| 6376 | CX3CL1 | q13 | 55,963,911 | 55,976,455 |
| 184  25242   7.29 |  |
| 1012 | CDH13 | q23.3 | 81,218,078 | 82,387,698 |
| qter: 79 |  |

# Chromosome 17 (9 loci)

Overall | jones-supp-luminal | jones-supp-myoepithelial | Locus ID | Symbol | Cytoband | Start | End || pter: 137 |  |  | 57555 | NLGN2 | p13.1 | 7,252,225 | 7,263,903 |
| 6  65   93.00 | 6  65   93.00 |
| 5430 | POLR2A | p13.1 | 7,328,421 | 7,358,653 |
| 4  58   68.71 | 4  58   68.71 |
| 1973 | EIF4A1 | p13.1 | 7,416,867 | 7,422,674 |
| 134  15560   8.61 | 389  30385   12.80 |
| 3965 | LGALS9 | q11.2 | 22,982,362 | 23,000,707 |
| 254  14807   17.15 | 362  20384   17.76 |
| 284119 | PTRF | q21.2 | 37,808,000 | 37,828,800 |
| 107  5556   19.26 | 146  7660   19.06 |
| 79170 | MGC11242 | q21.32 | 43,384,333 | 43,390,109 |
| 38  2099   18.11 | 214  26866   7.97 |
| 3675 | ITGA3 | q21.33 | 45,488,729 | 45,522,843 |
| 5  94   53.41 | 5  94   53.41 |
| 1277 | COL1A1 | q21.33 | 45,616,455 | 45,633,992 |
| 169  24622   6.86 |  |
| 9368 | SLC9A3R1 | q25.1 | 70,256,378 | 70,277,089 |
| qter: 147 |  |

# Chromosome 18 (2 loci)

Overall | jones-supp-luminal | jones-supp-myoepithelial | Locus ID | Symbol | Cytoband | Start | End || pter: 39 |  |  | 3613 | IMPA2 | p11.21 | 11,971,454 | 12,020,876 |
| 150  47274   3.17 | 150  47274   3.17 |
| 5268 | SERPINB5 | q21.33 | 59,295,198 | 59,323,297 |
| qter: 43 |  |

# Chromosome 19 (12 loci)

Overall | jones-supp-luminal | jones-supp-myoepithelial | Locus ID | Symbol | Cytoband | Start | End || pter: 0 |  |  | 8612 | PPAP2C | p13.3 | 232,045 | 242,435 |
| 13  385   33.77 | 13  385   33.77 |
| 10272 | FSTL3 | p13.3 | 627,416 | 634,389 |
| 65  1793   36.25 | 65  1793   36.25 |
| 4616 | GADD45B | p13.3 | 2,427,256 | 2,429,255 |
| 88  3935   22.36 | 179  8168   21.91 |
| 8570 | KHSRP | p13.3 | 6,364,358 | 6,375,805 |
| 90  4221   21.32 | 332  28014   11.85 |
| 57153 | CTL2 | p13.2 | 10,597,228 | 10,616,232 |
| 241  23774   10.14 | 702  45537   15.42 |
| 7386 | UQCRFS1 | q12 | 34,390,006 | 34,395,954 |
| 27  5152   5.24 | 27  5152   5.24 |
| 2821 | GPI | q13.11 | 39,547,908 | 39,583,076 |
| 98  4389   22.33 | 98  4389   22.33 |
| 3963 | LGALS7 | q13.2 | 43,971,693 | 43,974,232 |
| 31  1043   29.73 | 31  1043   29.73 |
| 2091 | FBL | q13.2 | 45,016,937 | 45,028,813 |
| 167  6941   24.06 | 167  6941   24.06 |
| 6510 | SLC1A5 | q13.32 | 51,969,981 | 51,983,653 |
| 133  4170   31.89 | 339  11764   28.82 |
| 5653 | KLK6 | q13.41 | 56,153,699 | 56,164,741 |
| 205  7583   27.03 |  |
| 10155 | TRIM28 | q13.43 | 63,747,647 | 63,753,894 |
| qter: 4 |  |

# Chromosome 20 (5 loci)

Overall | jones-supp-luminal | jones-supp-myoepithelial | Locus ID | Symbol | Cytoband | Start | End || pter: 69 |  |  | 5621 | PRNP | p13 | 4,615,068 | 4,630,233 |
| 27  5936   4.55 | 27  5936   4.55 |
| 182 | JAG1 | p12.2 | 10,566,333 | 10,602,590 |
| 167  24977   6.69 | 167  24977   6.69 |
| 10904 | BLCAP | q11.23 | 35,579,233 | 35,589,678 |
| 50  7766   6.44 |  |
| 8785 | MATN4 | q13.12 | 43,355,500 | 43,367,645 |
| 5  164   30.46 | 5  164   30.46 |
| 10406 | WFDC2 | q13.12 | 43,531,807 | 43,543,585 |
| qter: 172 |  |

# Chromosome 21 (2 loci)

Overall | jones-supp-luminal | jones-supp-myoepithelial | Locus ID | Symbol | Cytoband | Start | End || pter: 104 |  |  | 873 | CBR1 | q22.12 | 36,364,190 | 36,367,332 |
| 31  5094   6.09 |  |
| 25825 | BACE2 | q22.3 | 41,461,597 | 41,570,394 |
| qter: 93 |  |

# Chromosome 22 (2 loci)

Overall | jones-supp-luminal | jones-supp-myoepithelial | Locus ID | Symbol | Cytoband | Start | End || pter: 156 |  |  | 23481 | PES1 | q12.2 | 29,297,166 | 29,312,448 |
| 25  2209   11.32 | 25  2209   11.32 |
| 7078 | TIMP3 | q12.3 | 31,521,361 | 31,583,581 |
| qter: 207 |  |

# Chromosome X (2 loci)

Overall | jones-supp-luminal | jones-supp-myoepithelial | Locus ID | Symbol | Cytoband | Start | End || pter: 177 |  |  | 7076 | TIMP1 | p11.3–p11.23 | 47,198,084 | 47,202,441 |
| 36  1582   22.75 | 36  1582   22.75 |
| 5355 | PLP2 | p11.23 | 48,784,643 | 48,787,808 |
| qter: 470 |  |
